# Shadows of quantum machine learning

**DOI:** 10.1038/s41467-024-49877-8

**Published:** 2024-07-06

**Authors:** Sofiene Jerbi, Casper Gyurik, Simon C. Marshall, Riccardo Molteni, Vedran Dunjko

**Affiliations:** 1 Institute for Theoretical Physics, University of Innsbruck, Innsbruck, Austria; 2 Dahlem Center for Complex Quantum Systems, Freie Universität Berlin, Berlin, Germany; 3 applied Quantum algorithms (aQa), Leiden University, Leiden, The Netherlands

**Keywords:** Quantum information, Computer science, Information theory and computation

## Abstract

Quantum machine learning is often highlighted as one of the most promising practical applications for which quantum computers could provide a computational advantage. However, a major obstacle to the widespread use of quantum machine learning models in practice is that these models, even once trained, still require access to a quantum computer in order to be evaluated on new data. To solve this issue, we introduce a class of quantum models where quantum resources are only required during training, while the deployment of the trained model is classical. Specifically, the training phase of our models ends with the generation of a ‘shadow model’ from which the classical deployment becomes possible. We prove that: (i) this class of models is universal for classically-deployed quantum machine learning; (ii) it does have restricted learning capacities compared to ‘fully quantum’ models, but nonetheless (iii) it achieves a provable learning advantage over fully classical learners, contingent on widely believed assumptions in complexity theory. These results provide compelling evidence that quantum machine learning can confer learning advantages across a substantially broader range of scenarios, where quantum computers are exclusively employed during the training phase. By enabling classical deployment, our approach facilitates the implementation of quantum machine learning models in various practical contexts.

## Abstract (web summary)

Quantum machine learning faces applicability challenges as quantum computers are needed for both training and evaluation of trained models. This study explores models that can be quantumly trained but classically evaluated, highlighting their limits compared to fully quantum models and their advantages over classical ones.

## Introduction

Quantum machine learning is a rapidly growing field^1–3^ driven by its potential to achieve quantum advantages in practical applications. A particularly interesting approach to make quantum machine learning applicable in the near term is to develop learning models based on parametrized quantum circuits^4–6^. Indeed, such quantum models have already been shown to achieve good learning performance in benchmarking tasks, both in numerical simulations^7–11^ and on actual quantum hardware^12–15^. Moreover, based on widely believed cryptography assumptions, these models also hold the promise to solve certain learning tasks that are intractable for classical algorithms^16,17^, including predicting ground state properties of highly-interacting quantum systems^18^.

Despite these advances, quantum machine learning is facing a major obstacle for its use in practice. A typical workflow of a machine learning model involved, e.g., in driving autonomous vehicles, is divided into: (i) a training phase, where the model is trained, typically using training data or by reinforcement; followed by (ii) a deployment phase, where the trained model is evaluated on new input data. For quantum machine learning models, both of these phases require access to a quantum computer. But given that in many practical machine learning applications, the trained model is meant for a widespread deployment, the current scarcity of quantum computing access dramatically reduces the applicability of quantum machine learning. One way of addressing this problem is by generating shadow models out of quantum machine learning models. That is, we propose inserting a shadowing phase between the training and deployment, where a quantum computer is used to collect information on the quantum model. Then a classical computer can use this information to evaluate the model on new data during the deployment phase.

The conceptual idea of generating shadows of quantum models was already proposed by Schreiber et al.^19^, albeit under the terminology of classical surrogates. In that work, as well as in that of Landman et al.^20^, the authors make use of the general expression of quantum models as trigonometric polynomials^21^ to learn the Fourier representation of trained models and evaluate them classically on new data. However, these works also suggest that a classical model could potentially be trained directly on the training data and achieve the same performance as the shadow model, thus circumventing the need for a quantum model in the first place. This raises the concern that all quantum models that are compatible with a classical deployment would also lose all quantum advantage, hence severely limiting the prospects for a widespread use of quantum machine learning.

Therefore, two natural open questions are raised:

1. Can shadow models achieve a quantum advantage over entirely classical (classically trained and classically evaluated) models?
2. Do there exist quantum models that do not admit efficiently evaluatable shadow models?

In this work, we resolve both of these key open questions. We propose a general definition for shadow models, rooted in the fundamental idea that quantum machine learning models can be universally expressed as linear models^22^. This formulation of shadow models allows us to leverage various results and techniques from quantum information theory for the analysis of this model class. From a practical perspective, employing shadow tomography techniques^23–26^ allows to easily construct diverse shadow models that will resonate with the practitioners of quantum machine learning. Furthermore, in our exploration of the computational capabilities of shadow models, we find them to capture a distinct computational class. Specifically, we demonstrate that, under widely believed cryptography assumptions, there exist learning tasks where shadow models exhibit a provable quantum advantage over fully classical models. However, contrary to this advantage, we also establish that there exist quantum models that are strictly more powerful than the class of shadow models, based on common assumptions in complexity theory.

For ease of exposition, we will first adhere to a working definition of a shadow model as a model that is trained on a quantum computer, but can be evaluated classically on new input data with the help of information generated by a quantum computer (i.e., quantum-generated advice) that is independent of the new data. We will (informally) call a model “shadowfiable" if there exists a method of turning it into a shadow model. In the Section “General shadow models”, we will make our definitions more precise.

## Results

### The flipped model

**Fig. 1:**
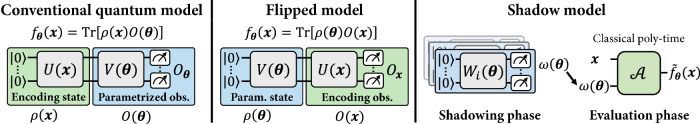
Quantum and shadow models. (left) Conventional quantum models can be expressed as inner products between a data-encoding quantum state *ρ*(***x***) and a parametrized observable *O*(***θ***). The resulting linear model ${f}_{{{{{{{{\boldsymbol{\theta }}}}}}}}}({{{{{{{\boldsymbol{x}}}}}}}})={{{{{{{\rm{Tr}}}}}}}}[\rho ({{{{{{{\boldsymbol{x}}}}}}}})O({{{{{{{\boldsymbol{\theta }}}}}}}})]$ naturally corresponds to a quantum computation, depicted here. (middle) We define flipped models ${f}_{{{{{{{{\boldsymbol{\theta }}}}}}}}}({{{{{{{\boldsymbol{x}}}}}}}})={{{{{{{\rm{Tr}}}}}}}}[\rho ({{{{{{{\boldsymbol{\theta }}}}}}}})O({{{{{{{\boldsymbol{x}}}}}}}})]$ as quantum linear models where the role of the quantum state *ρ*(***θ***) and the observable *O*(***x***) is flipped compared to conventional models. (right) Flipped models are associated to natural shadow models: one can use techniques from shadow tomography to construct a classical representation $\hat{\rho }({{{{{{{\boldsymbol{\theta }}}}}}}})$ of the parametrized state *ρ*(***θ***) (during the shadowing phase), such that, for encoding observables *O*(***x***) that are classically representable (e.g., linear combinations of Pauli observables), $\hat{\rho }({{{{{{{\boldsymbol{\theta }}}}}}}})$ can be used by a classical algorithm to evaluate the model *f*_***θ***_(***x***) on new input data (during the evaluation phase). More generally, a shadow model is defined by (i) a shadowing phase where a (bit-string) advice *ω*(***θ***) is generated by the evaluation of multiple quantum circuits *W*_1_(***θ***), …, *W*_*M*_(***θ***), and (ii) an evaluation phase where this advice is used by a classical algorithm ${{{{{{{\mathcal{A}}}}}}}}$, along with new input data ***x*** to evaluate their labels ${\widetilde{f}}_{{{{{{{{\boldsymbol{\theta }}}}}}}}}({{{{{{{\boldsymbol{x}}}}}}}})$. In the Section “General shadow models”, we show that under this general definition, all shadow models are shadows of flipped models.

The construction of our shadow models starts from a simple yet key observation: all standard quantum machine learning models for supervised learning can be expressed as linear models^22^. To delve into this claim, we first draw upon early works that utilized parametrized quantum circuits in machine learning^7,12^. These works proposed quantum models that are naturally expressed as linear functions of the form

1 $${f}_{{{{{{{{\boldsymbol{\theta }}}}}}}}}(x)={{{{{{{\rm{Tr}}}}}}}}[\rho (x)O({{{{{{{\boldsymbol{\theta }}}}}}}})]$$

where *ρ*(***x***) are quantum states that encode classical data ${{{{{{{\boldsymbol{x}}}}}}}}\in {{{{{{{\mathcal{X}}}}}}}}$ and *O*(***θ***) are parametrized observables whose inner product with *ρ*(***x***) defines *f*_***θ***_(***x***) (see Fig. 1). In a regression task, one would use such a model to assign a real-valued label to an input ***x***, while in classification tasks, one would additionally apply, e.g., a sign function, to discretize its output into a class. From a circuit picture, such models can be evaluated on a quantum computer by: (i) preparing an initial state *ρ*_0_, e.g., $\left\vert 0\right\rangle \, {\left\langle 0\right\vert }^{\otimes n}$, (ii) evolving it under a data-dependent circuit *U*(***x***), (iii) followed by a variational circuit *V*(***θ***), (iv) before finally measuring the expectation value of a Hermitian observable *O*. Together, steps (i) and (ii) define

2 $$\rho (x)=U(x){\rho }_{0}{U}^{{{{\dagger}}} }(x),$$

while steps (iii) and (iv) define

3 $$O({{{{{{{\boldsymbol{\theta }}}}}}}})={V}^{{{{\dagger}}} }({{{{{{{\boldsymbol{\theta }}}}}}}})O{V}({{{{{{{\boldsymbol{\theta }}}}}}}}).$$

Since the early works, it is known that quantum linear models also capture quantum kernel models as a special case^27^, simply by making *O*(***θ***) directly dependent on the training data of the learning task. Perhaps more surprisingly, quantum linear models can also encompass more general data re-uploading models, composed of several layers of data encoding and variational processing *U*_1_(***x***)*V*_1_(***θ***)*U*_2_(***x***)… Indeed, data re-uploading models can be mapped to linear models through circuit transformations (e.g., gate teleportation) that relocate all data-encoding gates to the first layer of the circuit^22^.

### Flipped model definition

The definition of a quantum linear model in Eq. (1) can in general accommodate any pair of Hermitian operators in place of *ρ*(***x***), *O*(***θ***). However, due to how these models are evaluated on a quantum computer, one commonly works under the constraint that *ρ*(***x***) defines a quantum state (i.e., a positive semi-definite operator with unit trace). Indeed, from an operational perspective, *ρ*(***x***) must be physically prepared on a quantum device before being measured with respect to the observable *O*(***θ***) (which only needs to be Hermitian in order to be a valid observable).

For reasons that will become clearer from the shadowing perspective, we define a so-called flipped model, where we flip the role of *ρ*(***x***) and *O*(***θ***). That is, we consider

4 $${f}_{{{{{{{{\boldsymbol{\theta }}}}}}}}}({{{{{{{\boldsymbol{x}}}}}}}})={{{{{{{\rm{Tr}}}}}}}}[\rho ({{{{{{{\boldsymbol{\theta }}}}}}}})O({{{{{{{\boldsymbol{x}}}}}}}})]$$

where *ρ*(***θ***) is a parametrized quantum state and *O*(***x***) is an observable that encodes the data and can take more general forms than Eq. (3) as we will see next. This model also corresponds to a straightforward quantum computation as *ρ*(***θ***) can be physically prepared before being measured with respect to *O*(***x***).

A simple example of flipped model is for instance defined by:

5 $$\rho ({{{{{{{\boldsymbol{\theta }}}}}}}})=V({{{{{{{\boldsymbol{\theta }}}}}}}}){\rho }_{0}{V}^{{{{\dagger}}} }({{{{{{{\boldsymbol{\theta }}}}}}}})\quad\&\quad O({{{{{{{\boldsymbol{x}}}}}}}})={\sum}_{j=1}^{m}{w}_{j}({{{{{{{\boldsymbol{x}}}}}}}}){P}_{j}$$

for an initial state *ρ*_0_, a variational circuit *V*(***θ***), and a collection of Pauli observables ${\{{P}_{j}\}}_{j=1}^{m}$ weighted by data-dependent weights ${w}_{j}({{{{{{{\boldsymbol{x}}}}}}}})\in {\mathbb{R}}$. One can evaluate this model by repeatedly preparing *ρ*(***θ***) on a quantum computer, measuring it in a Pauli basis specified by a *P*_*j*_, and weighting the outcome by *w*_*j*_(***x***). For other examples of flipped models, see Supplementary Section 1.

As opposed to conventional quantum linear models, flipped models are well-suited to construct shadow models. Since the variational operators *ρ*(***θ***) are quantum states, one can straightforwardly use techniques from shadow tomography^23^ to construct classical shadows $\hat{\rho }({{{{{{{\boldsymbol{\theta }}}}}}}})$ of these states. What we call classical shadows $\hat{\rho }({{{{{{{\boldsymbol{\theta }}}}}}}})$ here are collections of measurement outcomes obtained from copies of *ρ*(***θ***) that can be used to classically approximate expectation values of certain observables *O* (for a certain restricted family). If we take these observables to be our data-dependent *O*(***x***), then we end up with a classical model ${\widetilde{f}}_{{{{{{{{\boldsymbol{\theta }}}}}}}}}({{{{{{{\boldsymbol{x}}}}}}}})$ that approximates our flipped model. Note here that one has total freedom on the classical shadow techniques they may use to define their shadow models, and a plethora of protocols have already been proposed in the literature^23–26^. But it is important to keep in mind that each of these protocols comes with its limitations, as it may restrict the class of states *ρ*(***θ***) or the class of observables *O*(***x***) for which an efficient and faithful shadow model can be constructed. By “efficient” we refer here to the number of measurements performed on *ρ*(***θ***) and the time complexity of estimating the expectation values of observables *O*(***x***) from these measurements. And by “faithful” we refer to the approximation error between the shadow model ${\widetilde{f}}_{{{{{{{{\boldsymbol{\theta }}}}}}}}}({{{{{{{\boldsymbol{x}}}}}}}})$ resulting from the shadow protocol and the original flipped model *f*_***θ***_(***x***). For instance, in the example of Eq. (5), we know that if all Pauli operators ${\{{P}_{j}\}}_{j=1}^{m}$ are *k*-local, then $\widetilde{{{{{{{{\mathcal{O}}}}}}}}}({3}^{k}{B}^{2}{\varepsilon }^{-2})$ measurements of *ρ*(***θ***), where $B\,=\,{\max }_{{{{{{{{\boldsymbol{x}}}}}}}}}{\sum }_{j=1}^{m}| {w}_{i}({{{{{{{\boldsymbol{x}}}}}}}})|$, are sufficient to guarantee ${\max }_{{{{{{{{\boldsymbol{x}}}}}}}}}\left\vert \right.\,{\widetilde{f}}_{{{{{{{{\boldsymbol{\theta }}}}}}}}}({{{{{{{\boldsymbol{x}}}}}}}})-{f}_{{{{{{{{\boldsymbol{\theta }}}}}}}}}({{{{{{{\boldsymbol{x}}}}}}}})\left\vert \right.\le \varepsilon$ with high probability. But for non-local Pauli operators (i.e., large *k*), this protocol becomes highly inefficient if we want to guarantee a low error *ε*.

Importantly, shadowfied flipped models are not limited to constructions based on classical shadow protocols. Given that the states *ρ*(***θ***) are not given to us a black-box (as is generally assumed in shadow tomography), one can use prior knowledge on these states to construct efficient shadowing procedure. For instance, if *ρ*(***θ***) is known to be a superposition of a tractable number of computational basis states, or well-approximated by a matrix product state (MPS) with low bond dimension, then efficient tomography protocols may be used^28^.

### Properties of flipped models

Flipped models are a stepping stone toward the claims of quantum advantage and “shadowfiability” that are the focus of this paper. Nonetheless, they constitute a newly introduced model, which is why it is useful to understand first how they relate to previous quantum models and what learning guarantees they can have.

Since conventional linear models of the form of Eq. (1) play a central role in quantum machine learning, we start by asking the question: when can these models be represented by (efficiently evaluatable) flipped models? That is, given a conventional model ${f}_{{{{{{{{\boldsymbol{\theta }}}}}}}}}({{{{{{{\boldsymbol{x}}}}}}}})\,=\,{{{{{{{\rm{Tr}}}}}}}}[\rho ({{{{{{{\boldsymbol{x}}}}}}}})O({{{{{{{\boldsymbol{\theta }}}}}}}})]$, can we construct a flipped model ${\widetilde{f}}_{{{{{{{{\boldsymbol{\theta }}}}}}}}}({{{{{{{\boldsymbol{x}}}}}}}})\,=\,{{{{{{{\rm{Tr}}}}}}}}[\rho ^{\prime} ({{{{{{{\boldsymbol{\theta }}}}}}}})O^{\prime} ({{{{{{{\boldsymbol{x}}}}}}}})]$ such that ${\widetilde{f}}_{{{{{{{{\boldsymbol{\theta }}}}}}}}}({{{{{{{\boldsymbol{x}}}}}}}})\,\approx \,{f}_{{{{{{{{\boldsymbol{\theta }}}}}}}}}({{{{{{{\boldsymbol{x}}}}}}}}),\forall {{{{{{{\boldsymbol{x}}}}}}}},{{{{{{{\boldsymbol{\theta }}}}}}}}$, and ${\widetilde{f}}_{{{{{{{{\boldsymbol{\theta }}}}}}}}}({{{{{{{\boldsymbol{x}}}}}}}})$ is as efficient to evaluate as *f*_***θ***_(***x***). Clearly, a conventional model *f*_***θ***_(***x***) for which the parametrized operator *O*(***θ***) is also a quantum state (i.e., a positive semi-definite trace-1 operator) is by definition also a flipped model. Therefore, a natural strategy to flip a conventional model is to transform its observable *O*(***θ***) into a quantum state $\rho ^{\prime} ({{{{{{{\boldsymbol{\theta }}}}}}}})$. This transformation involves dealing with the negative eigenvalues of *O*(***θ***), which can be taken into account using an auxiliary qubit, without overheads in the efficiency of evaluation (see Supplementary Section 2 for more details). More importantly, the transformation involves normalizing these eigenvalues, which affects the efficiency of evaluating the resulting flipped model. Indeed, the normalization factor *α* that results from normalizing *O*(***θ***) corresponds to its trace norm $\parallel O{\parallel }_{1}\,=\,{{{{{{{\rm{Tr}}}}}}}}\left[\sqrt{{O}^{2}}\right]$ and needs to be absorbed into the observable $O^{\prime} ({{{{{{{\boldsymbol{x}}}}}}}})\,=\,\alpha \rho ({{{{{{{\boldsymbol{x}}}}}}}})$ of the flipped model ${\widetilde{f}}_{{{{{{{{\boldsymbol{\theta }}}}}}}}}({{{{{{{\boldsymbol{x}}}}}}}})$ to guarantee ${\widetilde{f}}_{{{{{{{{\boldsymbol{\theta }}}}}}}}}({{{{{{{\boldsymbol{x}}}}}}}})\,=\,{f}_{{{{{{{{\boldsymbol{\theta }}}}}}}}}({{{{{{{\boldsymbol{x}}}}}}}})$. This directly impacts the spectral norm $\parallel O^{\prime} {\parallel }_{\infty }={\max }_{\left\vert \psi \right\rangle }{\langle O^{\prime} \rangle }_{\psi }=\alpha$ of the flipped model, and therefore the efficiency of its evaluation, as ${{{{{{{\mathcal{O}}}}}}}}(\parallel O^{\prime} {\parallel }_{\infty }^{2}/{\varepsilon }^{2})$ measurements of $\rho ^{\prime} ({{{{{{{\boldsymbol{\theta }}}}}}}})$ are needed in order to estimate ${\widetilde{f}}_{{{{{{{{\boldsymbol{\theta }}}}}}}}}({{{{{{{\boldsymbol{x}}}}}}}})$ to additive error *ε* (see Supplementary Section 2 for a derivation). Therefore, we end up showing that, for a conventional model *f*_***θ***_(***x***) acting on *n* qubits and with a bounded observable trace norm ∥*O*∥_1_ ≤ *α*, we can construct a flipped model acting on *m* = *n* + 1 qubits and with observable spectral norm $\parallel O^{\prime} {\parallel }_{\infty }=\alpha$.

Interestingly, in the relevant regime where the number of qubits *n*, *m* used by the linear models involved in this flipping is logarithmic in ∥*O*∥_1_ (e.g., where *O* is a Pauli observable and hence ∥*O*∥_1_ = 2^*n*^), we find that this requirement on the spectral norm $\parallel O^{\prime} {\parallel }_{\infty }$ of the resulting flipped model is unavoidable in the worst case, up to a logarithmic factor in ∥*O*∥_1_. We refer to Appendix Supplementary Section 2 for proofs of these statements and a more in-depth discussion.

Another property of interest in machine learning is the generalization performance of a learning model. That is, we want to bound the gap between the performance of the model on its training set (so-called training error) and its performance on the rest of the data space (or expected error). Such bounds have for instance been derived in terms of the number of encoding gates in the quantum model^29^, or the rank of its observable^30^. In the case of flipped model, we find instead a bound in terms of the number of qubits *n* and the spectral norm ∥*O*∥_*∞*_ of the observable. Since these quantities are operationally meaningful, this gives us a natural way of controlling the generalization performance of our flipped models. Stated informally, we find that if a flipped model achieves a small error ∣ *f*_***θ***_(***x***) − *f*(***x***)∣ ≤ *η* for all ***x*** in a training set of size *M*, then we only need *M* to scale as $\widetilde{\Omega }\left(\frac{n\parallel O{\parallel }_{\infty }^{2}}{\varepsilon {\eta }^{2}}\right)$ in order to guarantee a small expected error ∣ *f*_***θ***_(***x***) − *f*(***x***)∣ ≤ 2*η* with probability 1 − *ε* over the entire data distribution.

Note that the dependence on *n* and ∥*O*∥_*∞*_ is linear and quadratic, respectively, which means that we can afford a large number of qubits and a large spectral norm and still guarantee a good generalization performance. This is particularly relevant as the spectral norm is a controllable quantity, meaning we can easily fine-tune our models to perform well in training and generalize well. E.g., in the case of the model in Eq. (5), this spectral norm is bounded by ${\max }_{{{{{{{{\boldsymbol{x}}}}}}}}}{\sum }_{j=1}^{m}| {w}_{i}({{{{{{{\boldsymbol{x}}}}}}}})|$, which scales favourably with the number of qubits *n* if $m\in {{{{{{{\mathcal{O}}}}}}}}(\,{{\mbox{poly}}}\,(n))$ or if the vector ***w***(***x***) is sparse.

### Quantum advantage of a shadow model

We recall that we (informally) define shadow models as models that are trained on a quantum computer, but, after a shadowing procedure that collects information on the trained model, are evaluated classically on new input data. In this section, we consider the question of achieving a quantum advantage using such shadow models. It may seem at first sight that this question has a straightforward answer, which is “no": if the function learned by a model is classically computable, then there should be no room for a quantum advantage. However, as demonstrated in refs. ^17,31^, one can also achieve a quantum advantage based on so-called trap-door functions. These are functions that are believed to be hard to compute classically, unless given a key (or advice) that allows for an efficient classical computation. Notably, there exist trap-door functions where this key can be efficiently computed using a quantum computer, but not classically. This allows us to construct shadow models that make use of this quantum-generated key to compute an otherwise classically untractable function.

Similarly to related results showing a quantum advantage in machine learning with classical data^16,32^, we consider a learning task where the target function (i.e., the function generating the training data) is derived from cryptographic functions that are widely believed to be hard to compute classically. More precisely, we introduce a variant of the discrete cube root learning task^17^, which is hard to solve classically under a hardness assumption related to that of the RSA cryptosystem^33^. In this task, we consider target functions defined on ${{\mathbb{Z}}}_{N}=\{0,\ldots,N-1\}$ as

6 $${g}_{s}({{{{{{{\boldsymbol{x}}}}}}}})=\left\{\begin{array}{ll}1,\quad &\,{{\mbox{if}}}\,\root 3 \of {{{{{{{{\boldsymbol{x}}}}}}}}}\,{{\mbox{mod}}}\,N\in [s,s+\frac{N-1}{2}],\\ 0,\quad &\,{{\mbox{otherwise}}}\,\hfill\end{array}\right.$$

where *N* = *pq* is an *n*-bit integer, product of two primes *p*, *q* of the form $3k+2,\,3k^{\prime}+2$, such that the discrete cube root is properly defined as the inverse of the function ***y***^3^ mod *N*. These target functions are particularly appealing because of a number of interesting properties:

i. It is believed that given only ***x*** and *N* as input, computing $g({{{{{{{\boldsymbol{x}}}}}}}})=\root 3 \of {{{{{{{{\boldsymbol{x}}}}}}}}}\;{{{{{{\rm{mod}}}}}}}\,N$ with high probability of success over random draws of ***x*** and *N* is classically intractable. This assumption is known as the discrete cube root (DCR) assumption.
ii. On the other hand, computing ***x***^*a*^ mod *N* is classically efficient for any $a\in {{\mathbb{Z}}}_{N}$. For *a* = 3, this implies that *g*^−1^(***y***) = ***y***^3^ mod *N* is a one-way function, under the DCR assumption.
iii. The function $g({{{{{{{\boldsymbol{x}}}}}}}})=\root 3 \of {{{{{{{{\boldsymbol{x}}}}}}}}}\;{{\mbox{mod}}}\,N$ has a “trap-door”, in that there exists another way of computing it efficiently. For every *N* (as specified above), there exists a key $d\in {{\mathbb{Z}}}_{N}$ such that *g*(***x***) = ***x***^*d*^ mod *N*. Finding *d* is efficient quantumly by using Shor’s factoring algorithm^34^, but hard classically under the DCR assumption.

Observations (i) and (ii) can be leveraged to show that learning the functions *g*_*s*_ from examples is also intractable. Indeed, Alexi et al.^35^ showed that a classical algorithm that could faithfully capture a single bit *g*_*s*_(***x***) of the discrete cube root of ***x***, for even a 1/2 + 1/poly(*n*) fraction of all ${{{{{{{\boldsymbol{x}}}}}}}}\in {{\mathbb{Z}}}_{N}$, could also be used to reconstruct $g({{{{{{{\boldsymbol{x}}}}}}}}),\,\forall {{{{{{{\boldsymbol{x}}}}}}}}\in {{\mathbb{Z}}}_{N}$, with high probability of success. Since, from observation (ii), the training data for the learning algorithm can also be generated efficiently classically from *N*, a classical learner that learns *g*_*s*_(***x***) correctly for a 1/2 + 1/poly(*n*) fraction of all ${{{{{{{\boldsymbol{x}}}}}}}}\in {{\mathbb{Z}}}_{N}$ would then contradict the DCR assumption.

Observation (iii) allows us to define the following flipped model:

7 $${f}_{{{{{{{{\boldsymbol{\theta }}}}}}}}}({{{{{{{\boldsymbol{x}}}}}}}})	={{{{{{{\rm{Tr}}}}}}}}[\rho ({{{{{{{\boldsymbol{\theta }}}}}}}})O({{{{{{{\boldsymbol{x}}}}}}}})]\\ \rho ({{{{{{{\boldsymbol{\theta }}}}}}}})	=\left\vert d^{\prime},s^{\prime} \right\rangle \left\langle d^{\prime},s^{\prime} \right\vert \,\&\,O({{{{{{{\boldsymbol{x}}}}}}}})={\sum}_{d^{\prime},s^{\prime} }{\widehat{g}}_{d^{\prime},s^{\prime} }({{{{{{{\boldsymbol{x}}}}}}}})\left\vert d^{\prime},s^{\prime} \right\rangle \left\langle d^{\prime},s^{\prime} \right\vert .$$

That is, *ρ*(***θ***) (for ${{{{{{{\boldsymbol{\theta }}}}}}}}=(N,\, s^{\prime} )$) specifies candidates for the key $d^{\prime}$ and the parameter $s^{\prime}$ of interest, while *O*(***x***) uses that information to compute

8 $${\widehat{g}}_{d^{\prime},s^{\prime} }({{{{{{{\boldsymbol{x}}}}}}}})=\left\{\begin{array}{ll}1,\quad &\,{{\mbox{if}}}\,{{{{{{{{\boldsymbol{x}}}}}}}}}^{d^{\prime} }\,{{\mbox{mod}}}\,N\in [s^{\prime},s^{\prime}+\frac{N-1}{2}],\\ 0,\quad &\,{{\mbox{otherwise.}}}\,\hfill\end{array}\right.$$

The state $\rho ({{{{{{{\boldsymbol{\theta }}}}}}}})=\left\vert d,\, s^{\prime} \right\rangle \left\langle d,\, s^{\prime} \right\vert$ for the right key *d* can be prepared efficiently using Shor’s algorithm applied on *N* (provided with the training data). As for *O*(***x***), it simply processes classically a bit-string to compute ${\widehat{g}}_{d^{\prime},s^{\prime} }({{{{{{{\boldsymbol{x}}}}}}}})$ efficiently, which corresponds to *g*_*s*_(***x***) when $(d^{\prime},s^{\prime} )=(d,s)$. Finding an $s^{\prime}$ close to *s* is an easy task given training data and $d^{\prime}=d$. Since *ρ*(***θ***) is a computational basis state, this flipped model admits a trivial shadow model where a single computational basis measurement of *ρ*(***θ***) allows to evaluate *f*_***θ***_(***x***) classically for all ***x***. Therefore, we end up showing the following theorem:

#### Theorem 1

**(Quantum advantage (informal))**. There exists a learning task where a shadow model first trained using a quantum computer then evaluated classically on new input data, can achieve an arbitrarily good learning performance, while any fully classical model cannot do significantly better than random guessing, under the hardness of classically computing the discrete cube root.

In Supplementary Section 3, we formalize the statement of this result using the PAC framework and provide more details on the setting and the proofs.

### General shadow models

As mentioned at the start of this paper, shadow models are not limited to shadowfied flipped models, and the main alternative proposals are based on the Fourier representation of quantum models^19,20^. It is clear that Fourier models are defined very differently from flipped models, but one may wonder whether they nonetheless include shadowfied flipped models as a special case, or the other way around.

In this section, we first start by showing that there exist quantum models that admit shadow models (i.e., are shadowfiable) but cannot be shadowfied efficiently using a Fourier approach. This then motivates our proposal for a general definition of shadow models, and we show that, under this definition, all shadow models can be expressed as shadowfied flipped models. Finally, we show the existence of quantum models that are not shadowfiable at all under likely complexity theory assumptions.

### Shadow models beyond Fourier

An interesting approach to construct shadows of quantum models is based on their natural Fourier representation. It has been shown^21,36^ that quantum models can be expressed as generalized Fourier series of the form

9 $${f}_{{{{{{{{\boldsymbol{\theta }}}}}}}}}({{{{{{{\boldsymbol{x}}}}}}}})=\mathop{\sum}_{{{{{{{{\boldsymbol{\omega }}}}}}}}\in \Omega }{c}_{{{{{{{{\boldsymbol{\omega }}}}}}}}}({{{{{{{\boldsymbol{\theta }}}}}}}}){e}^{-i{{{{{{{\boldsymbol{\omega }}}}}}}}\cdot {{{{{{{\boldsymbol{x}}}}}}}}}$$

where the accessible frequencies Ω only depend on properties of the encoding gates used by the model (notably the number of encoding gates and their eigenvalues). Since these frequencies can easily be read out from the circuit, one can proceed to form a shadow model by estimating their associated coefficients *c*_***ω***_(***θ***) using queries of the quantum model *f*_***θ***_(***x***) at different values ***x*** and, e.g., a Fourier transform^19^. Given a good approximation of these coefficients, one can then compute estimates of *f*_***θ***_(***x***) for arbitrary new inputs ***x***. We will refer to such a shadowing approach that considers the quantum model as a black-box, aside from the knowledge of its Fourier spectrum, as the Fourier shadowing approach.

Although we will be explicit about this in the next subsection, we will consider a shadowing procedure to be successful, if, with high probability, the resulting shadow model agrees with the original model on all inputs, i.e.,

10 $${\max}_{{{{{{{{\boldsymbol{x}}}}}}}}\in {{{{{{{\mathcal{X}}}}}}}}}\left\vert \right.\,{f}_{{{{{{{{\boldsymbol{\theta }}}}}}}}}({{{{{{{\boldsymbol{x}}}}}}}})-{\widetilde{f}}_{{{{{{{{\boldsymbol{\theta }}}}}}}}}({{{{{{{\boldsymbol{x}}}}}}}})\left\vert \right.\le \varepsilon,$$

for a specified *ε* ≥ 0. We want the shadowing procedure to be successful independently of the data distribution under which the model should be trained, which justifies this definition. We discuss this point further in Supplementary Section 4.

We show that the Fourier shadowing approach can suffer from an exponential sample complexity in the dimension of the input data ***x***, making it intractable for high-dimensional input spaces. To see this, consider the linear model:

11 $${f}_{{{{{{{{\boldsymbol{y}}}}}}}}}({{{{{{{\boldsymbol{x}}}}}}}})	={{{{{{{\rm{Tr}}}}}}}}[\rho ({{{{{{{\boldsymbol{x}}}}}}}})O({{{{{{{\boldsymbol{y}}}}}}}})]\\ \rho ({{{{{{{\boldsymbol{x}}}}}}}})	={\bigotimes}_{i=1}^{n}{R}_{Y}({x}_{i})\left\vert 0\right\rangle \left\langle 0\right\vert {R}_{Y}^{{{{\dagger}}} }({x}_{i})\,\&\,O({{{{{{{\boldsymbol{y}}}}}}}})=\left\vert {{{{{{{\boldsymbol{y}}}}}}}}\right\rangle \left\langle {{{{{{{\boldsymbol{y}}}}}}}}\right\vert .$$

for ${{{{{{{\boldsymbol{x}}}}}}}}\in {{\mathbb{R}}}^{n}$ and ***y*** ∈ {0, 1}^⊗*n*^. Let us first restrict our attention to the domain ***x*** ∈ {0, *π*}^*n*^. It is quite clear that on this domain, *f*_***y***_(***x***) = *δ*_***x***/*π*,***y***_ plays the role of a database search oracle, where the database has 2^*n*^ elements and a unique marked element ***y***. From lower bounds on database search, we know that Ω(2^*n*^) calls to this oracle are needed to find ***y***^37^. This implies that a Fourier shadowing approach would require Ω(2^*n*^) calls to *f*_***y***_(***x***) = *δ*_***x***/*π*,***y***_ in order to guarantee ${\max}_{{{{{{{{\boldsymbol{x}}}}}}}}\in {{{{{{{\mathcal{X}}}}}}}}}\left\vert \right.\, {\widetilde{f}}_{{{{{{{{\boldsymbol{\theta }}}}}}}}}({{{{{{{\boldsymbol{x}}}}}}}})-{f}_{{{{{{{{\boldsymbol{\theta }}}}}}}}}({{{{{{{\boldsymbol{x}}}}}}}})\left\vert \right.\le 1/4$. In Supplementary Section 4, we explain how this result can be generalized to the full domain ${{{{{{{\boldsymbol{x}}}}}}}}\in {{\mathbb{R}}}^{n}$, and we relate this bound on the sample complexity to the Fourier decomposition of the model.

On the other hand, note that the flipped model associated to *f*_***y***_(***x***) allows for a straightforward shadowing procedure. Indeed, by preparing *O*(***y***) and measuring it in the computational basis, one straightforwardly obtains ***y*** and can therefore classically compute the expectation value of any tensor product observable *ρ*(***x***) as specified by Eq. (11). Therefore, we have shown that there exist shadowfiable models that are not efficiently Fourier-shadowfiable, i.e., for which a shadowing procedure based solely on the knowledge of their Fourier spectrum and on black-box queries has query complexity that is exponential in the input dimension.

### All shadow models are shadows of flipped models

We give a general definition of shadow models that can encompass all methods that have been proposed to generate them. In contrast to the definition of classical surrogates proposed by Schreiber et al.^19^, we give explicit definitions for the shadowing and evaluation phases of shadow models which makes explicit the need for a quantum computer in the shadowing phase. Indeed, as mentioned in the introduction, the term “classical surrogate” has been used to describe both a classically evaluatable model obtained from a quantum shadowing procedure and a fully classical model trained directly on the data. We want to avoid this confusion in the definition of shadow models. We view a general shadowing phase as the generation of advice that can be used to classically evaluate a quantum model. This advice is generated by the execution of quantum circuits that may or may not depend on the (trained) quantum circuit from the training phase. For instance, when we shadowfy a flipped model, we simply prepare the parametrized states *ρ*(***θ***) and use (randomized) measurements to generate an operationally meaningful classical description. In the case of Fourier shadowing, this advice is instead generated by evaluations of the quantum model *f*_***θ***_(***x***) for different inputs ${{{{{{{\boldsymbol{x}}}}}}}}\in {{\mathbb{R}}}^{d}$ that are rich enough to learn the Fourier coefficients of this model. We propose the following definition:

#### Definition 2

**(General shadow model)**. Let *W*_1_(***θ***), . . . , *W*_*M*_(***θ***) be a sequence of ${{{{{{{\mathcal{O}}}}}}}}(\,{{\mbox{poly}}}\,(m))$-time quantum circuits applied on all-zero states ${\left\vert 0\right\rangle }^{\otimes m}$, and that can potentially be chosen adaptively. Call *ω*(***θ***) = (*ω*_1_(***θ***), …, *ω*_*M*_(***θ***)) the outcomes of measuring the output states of these circuits in the computational basis. A general shadow model is defined as:

12 $${f}_{{{{{{{{\boldsymbol{\theta }}}}}}}}}({{{{{{{\boldsymbol{x}}}}}}}})={{{{{{{\mathcal{A}}}}}}}}({{{{{{{\boldsymbol{x}}}}}}}},\omega ({{{{{{{\boldsymbol{\theta }}}}}}}}))$$

where ${{{{{{{\mathcal{A}}}}}}}}$ is a classical ${{{{{{{\mathcal{O}}}}}}}}(\,{{\mbox{poly}}}\,(M,m,d))$ time algorithm that processes the outcomes *ω*(***θ***) along with an input ${{{{{{{\boldsymbol{x}}}}}}}}\in {{\mathbb{R}}}^{d}$ to return the (real-valued) label *f*_***θ***_(***x***).

From this definition, a shadow model is a classically evaluatable model that uses quantum-generated advice. Crucially, this advice must be independent of the data points ***x*** we wish to evaluate the model on in the future. We distinguish the notion of a shadow model from that of a shadowfiable quantum model, that is a quantum model that admits a shadow model:

#### Definition 3

**(Shadowfiable model)**. A model *f*_***θ***_ acting on *n* qubits is said to be shadowfiable if, for *ε*, *δ* > 0, there exists a shadow model ${\widetilde{f}}_{{{{{{{{\boldsymbol{\theta }}}}}}}}}$ such that, with probability 1 − *δ* over the quantum generation of the advice *ω*(***θ***) (i.e., the shadowing phase), the shadow model satisfies?

13 $${\max}_{{{{{{{{\boldsymbol{x}}}}}}}}\in {{{{{{{\mathcal{X}}}}}}}}}| \,{f}_{{{{{{{{\boldsymbol{\theta }}}}}}}}}({{{{{{{\boldsymbol{x}}}}}}}})| -{\widetilde{f}}_{{{{{{{{\boldsymbol{\theta }}}}}}}}}({{{{{{{\boldsymbol{x}}}}}}}})\le \varepsilon,$$

and uses $m,M\in {{{{{{{\mathcal{O}}}}}}}}(\,{{\mbox{poly}}}\,(n,1/\varepsilon,1/\delta ))$ qubits and circuits to generate its advice *ω*(***θ***).

While we have seen that there exist shadowfiable models that cannot be shadowfied efficiently using a Fourier approach, we show that all shadowfiable models as defined above can be approximated by shadowfiable flipped models.

#### Lemma 4

**(Flipped models are shadow-universal)**. All shadowfiable models as defined in Defs. 2 and 3 can be approximated by flipped models ${f}_{{{{{{{{\boldsymbol{\theta }}}}}}}}}({{{{{{{\boldsymbol{x}}}}}}}})\,=\,{{{{{{{\rm{Tr}}}}}}}}[\rho ({{{{{{{\boldsymbol{\theta }}}}}}}})O({{{{{{{\boldsymbol{x}}}}}}}})]$ with the guarantee that computational basis measurements of *ρ*(***θ***) and efficient classical post-processing can be used to evaluate *f*_***θ***_(***x***) to good precision with high probability.

This result is essentially based on the observation that the evaluation of a general shadow model as defined in Def. 2 can be done entirely coherently. Instead of classically running the algorithm ${{{{{{{\mathcal{A}}}}}}}}$ using the random advice *ω*(***θ***), one can quantumly simulate this algorithm (using a reversible execution) and execute it on the coherent advice $\rho ({{{{{{{\boldsymbol{\theta }}}}}}}})=\left\vert \omega ({{{{{{{\boldsymbol{\theta }}}}}}}})\right\rangle \left\langle \omega ({{{{{{{\boldsymbol{\theta }}}}}}}})\right\vert$ generated by {*W*_1_(***θ***), . . . , *W*_*M*_(***θ***)} before the computational basis measurements. We refer to Supplementary Section 4 for a more detailed statement and proof.

### Not all quantum models are shadowfiable

**Fig. 2:**
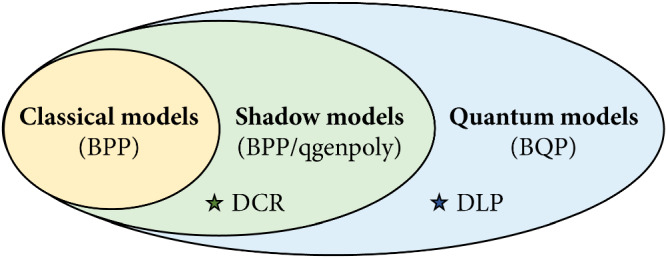
Separations between classical, shadow, and quantum models. Under the assumption that the discrete cube root (DCR) cannot be computed classically in polynomial time, we have a separation between shadow models (captured by the class BPP/qgenpoly) and classical models (in BPP). Under the assumption that there exist functions that can be computed in quantum polynomial time but not in classical polynomial time with the help of advice (i.e., BQP ⊄ P/poly), we have a separation between quantum models (universal for BQP) and shadow models (BPP/qgenpoly). A candidate function for this separation is the discrete logarithm (DLP).

From the discrete cube root learning task, we already understand that a learning separation can be established between classical and shadowfiable models. We would also like to understand whether a learning separation exists between shadowfiable models and general quantum models, or equivalently, whether all quantum models are shadowfiable. We show that this also is not the case, under widely believed assumptions (see Fig. 2).

#### Theorem 5

**(Not all shadowfiable)**. Under the assumption that BQP ⊄ P/poly, there exist quantum models, i.e., models in BQP, that are not shadowfiable, i.e., that are not in BPP/qgenpoly.

We start by noting that shadow models can be characterized by a complexity class we define as BPP/qgenpoly, which stands for “Bounded-error Probabilistic Polynomial-time with quantumly generated (polynomial-time) advice of polynomial size”. This class contains all functions that can be computed efficiently classically with the help of polynomially-sized advice generated efficiently by a quantum computer. This class is trivially contained in the standard class BPP/poly, which doesn’t have any constraint on how the advice is generated and can be derandomized to P/poly (i.e., BPP/poly = P/poly^38^). Note however that BPP/qgenpoly constitutes a physically relevant class, since it only contains problems that can be solved efficiently by classical and quantum computers, as opposed to P/poly, which contains undecidable problems, such as a version of the halting problem. We refer to Supplementary Section 1 for formal definitions of these complexity classes, and an in-depth discussion.

On the other hand, it is easy to show that quantum models (more precisely quantum linear models) can also represent any function in BQP, i.e., all functions that are efficiently computable on a quantum computer. For this, one simply takes a simple encoding of an *n*-bit input ***x***:

14 $$\rho ({{{{{{{\boldsymbol{x}}}}}}}}) \,=\, {\bigotimes}_{i=1}^{n}{X}_{i}^{{x}_{i}}\left\vert 0\right\rangle \left\langle 0\right\vert {X}_{i}^{{x}_{i}}$$

along with an observable

15 $${O}_{n}={U}_{n}^{{{{\dagger}}} }{Z}_{1}{U}_{n}$$

specified by an arbitrary *n*-qubit circuit *U*_*n*_ in BQP and the Pauli-*Z* operator applied on it first qubit. The resulting model ${f}_{n}({{{{{{{\boldsymbol{x}}}}}}}})={{{{{{{\rm{Tr}}}}}}}}[\rho ({{{{{{{\boldsymbol{x}}}}}}}}){O}_{n}]$ can then be used to decide any language in BQP.

Combining these two observations, we get that the proposition “all quantum models are shadowfiable” would imply that BQP ⊆ BPP/qgenpoly ⊆ P/poly, which violates the widely believed conjecture^39^ that BQP ⊈ P/poly (see Supplementary Section 4 for a formal proof). To give an example of candidates of non-shadowfiable quantum models, the discrete logarithm ${\log }_{g}x\,{{\mbox{mod}}}\,p$ (or even one bit of it) is provably in BQP but is not believed to be in P/poly. Therefore, a model that could be used to compute the discrete logarithm (e.g., the quantum model of Liu et al.^16^) is likely not shadowfiable.

## Discussion

In this work, we examined the class of quantumly trainable, classically evaluatable models we refer to as shadow models. Our analysis has shown that these models can be universally captured by a restricted family of quantum linear models, wherein data-encoding and variational operations are flipped compared to conventional quantum models. Furthermore, we demonstrated that shadow models belong to an intriguing complexity class, coined BPP/qgenpoly, exhibiting superiority over classical models (in BPP) but inferiority to fully quantum models (in BQP), based on prevalent complexity theory assumptions.

By presenting shadows models as flipped linear models, we illustrated how shadow tomography protocols could be applied straightforwardly to construct shadow models in practice. Yet, it is important to note a crucial distinction between a shadow tomography scenario and a shadow model: in the latter, one has control over the quantum state intended for shadowing. This distinction introduces new possibilities for devising ‘state-aware’ shadow tomography protocols aimed at constructing shadow models. This could potentially alleviate some of the limitations of current classical shadow protocols.

Considering our findings on learning separations, we identified a noteworthy characteristic of shadow models: their ability to quantumly compute useful advice for a classical evaluation algorithm, enabling them to tackle otherwise classically intractable tasks. The example we presented, based on trap-door functions, readily allows for such constructions, but it remains somewhat contrived. Exploring similar constructions for physically relevant problems, such as predicting ground state properties of complex quantum systems, would be an intriguing avenue for future research.

### Supplementary information

Supplementary Information

Peer Review File

## Data Availability

Data sharing is not applicable to this paper as no datasets were generated or analyzed during the current study.
