# Supplementary material for: Shadows of quantum machine learning
Source: Nat Commun. 2024 Jul 6;15:5676. doi: 10.1038/s41467-024-49877-8 (PMC11227511; doi:10.1038/s41467-024-49877-8)
Supplement: Supplementary file 1 — Supplementary Information [file 41467_2024_49877_MOESM1_ESM.pdf]

Supplementary Information for: Shadows of quantum machine learning

Sofiene Jerbi,[1,2] Casper Gyurik,[3] Simon C. Marshall,[3] Riccardo Molteni,[3] and Vedran Dunjko[3]

[1]*Institute for Theoretical Physics, University of Innsbruck, Austria*
[2]*Dahlem Center for Complex Quantum Systems, Freie Universität Berlin, Germany*
[3]*applied Quantum algorithms (aQa), Leiden University, The Netherlands*

1. Formal definitions

A. Linear models

Definition 1.1 (Conventional linear model)**.** *Let $U(\boldsymbol{x})$ be an encoding quantum circuit that is parametrized by input data $\boldsymbol{x} \in \mathbb{R}^d$, ρ_0 a fixed input quantum state (diagonal in the computational basis), $V(\boldsymbol{\theta})$ a variational quantum circuit parametrized by a vector $\boldsymbol{\theta} \in \mathbb{R}^p$ and $O = \sum_{i=1}^m w_i O_i$ an observable specified by a (trainable) linear combination of Hermitian matrices $\{O_i\}_{i=1}^m$. A conventional linear model is defined by the parametrized function:*

$$f_{\boldsymbol{\theta}}(\boldsymbol{x}) = \mathrm{Tr}[\rho(\boldsymbol{x})O(\boldsymbol{\theta})] \tag{1}$$

for $\rho(\boldsymbol{x}) = U(\boldsymbol{x})\rho_0 U^\dagger(\boldsymbol{x})$ and $O(\boldsymbol{\theta}) = V(\boldsymbol{\theta})OV^\dagger(\boldsymbol{\theta})$ (when the weights $\{w_i\}_{i=1}^m$ are also trainable, we include them in the parameters $\boldsymbol{\theta}$ of the model).

Definition 1.2 (Flipped model)**.** *Let $V(\boldsymbol{\theta})$ be a variational quantum circuit parametrized by a vector $\boldsymbol{\theta} \in \mathbb{R}^p$, ρ_0 a fixed input quantum state (diagonal in the computational basis), $U(\boldsymbol{x})$ an encoding quantum circuit that is parametrized by input data $\boldsymbol{x} \in \mathbb{R}^d$ and $O_{\boldsymbol{x}} = \sum_{i=1}^m w(\boldsymbol{x})_i O_i$ an observable specified by a linear combination of Hermitian matrices $\{O_i\}_{i=1}^m$, weighted by a data-dependent function $w : \mathbb{R}^d \to \mathbb{R}^m$. A flipped model is defined by the parametrized function:*

$$f_{\boldsymbol{\theta}}(\boldsymbol{x}) = \mathrm{Tr}[\rho(\boldsymbol{\theta})O(\boldsymbol{x})] \tag{2}$$

for $\rho(\boldsymbol{\theta}) = V(\boldsymbol{\theta})\rho_0 V^\dagger(\boldsymbol{\theta})$ and $O(\boldsymbol{x}) = U(\boldsymbol{x})O_{\boldsymbol{x}}U^\dagger(\boldsymbol{x})$.

B. Shadow models

Definition 1.3 (Shadow model)**.** *Let $\{W_1(\boldsymbol{\theta}), ..., W_M(\boldsymbol{\theta})\}$ be a sequence of m-qubit unitary circuits that are dependent on a parameter vector $\boldsymbol{\theta} \in \mathbb{R}^p$, and can potentially be chosen adaptively. We define the* quantum-generated *advice $\omega(\boldsymbol{\theta}) = (\omega_1(\boldsymbol{\theta}), \ldots, \omega_M(\boldsymbol{\theta}))$ as the measurement outcomes $\omega_i(\boldsymbol{\theta})$ obtained by measuring the states $W_i(\boldsymbol{\theta})\left|0\right\rangle^{\otimes m}$ in the computational basis (and a description of their associated circuits $W_i(\boldsymbol{\theta})$). A shadow model is defined as the parametrized function:*

$$f_{\boldsymbol{\theta}}(\boldsymbol{x}) = \mathcal{A}(\boldsymbol{x}, \omega(\boldsymbol{\theta})) \tag{3}$$

for $\mathcal{A}$ a classical $\mathcal{O}(poly(m, M, d))$-time algorithm that takes as input the advice $\omega(\boldsymbol{\theta})$, a data vector $\boldsymbol{x} \in \mathbb{R}^d$ and outputs a real-valued label $f_{\boldsymbol{\theta}}(\boldsymbol{x})$

Examples of shadow models:

- Take a flipped model $f_{\boldsymbol{\theta}}(\boldsymbol{x}) = \mathrm{Tr}[\rho(\boldsymbol{\theta})O(\boldsymbol{x})]$ for $\rho(\boldsymbol{\theta})$ a quantum state generated by a circuit $V(\boldsymbol{\theta})$ applied to $\left|0\right\rangle^{\otimes n}$ and $O(\boldsymbol{x}) = \sum_{i=1}^m w(\boldsymbol{x})_i P_i$ where $\{P_i\}_{i=1}^m$ are all k-local Pauli strings acting on n qubits.
  A simple shadow model associated to this flipped model consists in estimating all the expectation values $\langle P_i\rangle \approx \mathrm{Tr}[\rho(\boldsymbol{\theta})P_i]$ via repeated measurements of $\rho(\boldsymbol{\theta})$ in the eigenbasis of each of the $m = \binom{n}{k}3^k$ Pauli strings P_i, and taking their weighted combination $f_{\boldsymbol{\theta}}(\boldsymbol{x}) = \sum_{i=1}^m w(\boldsymbol{x})_i \langle P_i\rangle$. In this case, the unitary circuits $\{W_1(\boldsymbol{\theta}), ..., W_M(\boldsymbol{\theta})\}$ are simply obtained by $V(\boldsymbol{\theta})$ followed by a basis change unitary (corresponding to the Pauli basis of P_i). As for the classical algorithm $\mathcal{A}$, this is simply a collection of mean estimators that compute estimates $\langle P_i\rangle$ out of measurement outcomes, followed by the computation of a weighted sum. The number of measurements needed for this shadow model to guarantee $\left|\widetilde{f}_{\boldsymbol{\theta}}(\boldsymbol{x}) - f_{\boldsymbol{\theta}}(\boldsymbol{x})\right| \leq \varepsilon$, $\forall \boldsymbol{x} \in \mathbb{R}^d$ is $M \in \widetilde{\mathcal{O}}\left(\frac{m \max_{\boldsymbol{x}} \|w(\boldsymbol{x})\|_1^2}{\varepsilon^2}\right)$. Indeed, estimating each $\langle P_i\rangle$ to additive error $\frac{\varepsilon}{\max_{\boldsymbol{x}} \|w(\boldsymbol{x})\|_1}$ allows us to guarantee the desired total additive

error, and each of this estimates can be obtained using $\widetilde{\mathcal{O}}\left(\frac{\max_{\boldsymbol{x}} \|w(\boldsymbol{x})\|_1^2}{\varepsilon^2}\right)$ samples.

A more interesting shadow model relies on Pauli classical shadows [1] where random Pauli measurements are used to construct $\omega(\boldsymbol{\theta})$. Median-of-mean estimators then use these measurement outcomes to compute empirical estimates $\hat{\rho}(\boldsymbol{\theta})$ of $\rho(\boldsymbol{\theta})$ and approximate all expectation values $\mathrm{Tr}[\rho(\boldsymbol{\theta})P_i]$. The advantage of this shadow model is that it requires $M \in \widetilde{\mathcal{O}}\left(\frac{3^k \max_{\boldsymbol{x}} \|w(\boldsymbol{x})\|_1^2}{\varepsilon^2}\right)$ measurements for the same guarantees as the shadow model above, which constitutes savings of a factor $\widetilde{\mathcal{O}}\left(\binom{n}{k}\right)$.

- Another interesting flipped model that can be turned into a shadow model: $f_{\boldsymbol{\theta}}(\boldsymbol{x}) = \mathrm{Tr}[\rho(\boldsymbol{\theta})O(\boldsymbol{x})]$ for $\rho(\boldsymbol{\theta})$ arbitrarily defined and $O(\boldsymbol{x}) = |\psi(\boldsymbol{x})\rangle\langle\psi(\boldsymbol{x})|$, for some pure states $|\psi(\boldsymbol{x})\rangle$. Given that, $\mathrm{Tr}\left[O(\boldsymbol{x})^2\right] = 1,\ \forall \boldsymbol{x} \in \mathbb{R}^d$, then Clifford classical shadows [1] allow to construct a representation $\omega(\boldsymbol{\theta})$ of $\rho(\boldsymbol{\theta})$ that guarantees $\left|\widetilde{f}_{\boldsymbol{\theta}}(\boldsymbol{x}) - f_{\boldsymbol{\theta}}(\boldsymbol{x})\right| \leq \varepsilon,\ \forall \boldsymbol{x} \in \mathbb{R}^d$ using only $M \in \widetilde{\mathcal{O}}\left(\frac{1}{\varepsilon^2}\right)$ measurements. However, for this estimation to be computationally efficient, the states $|\psi(\boldsymbol{x})\rangle$ need to be stabilizer states, or be generated by few (i.e., $\mathcal{O}(\log(n))$) non-Clifford gates [2].

- Consider the model $f_{\boldsymbol{\theta}}(\boldsymbol{x}) = \sum_{i=1}^m w(\boldsymbol{x})_i \,\mathrm{Tr}[\rho(\boldsymbol{\theta})P_i]^2$ for $m = 4^n$, i.e., $\{P_i\}_{i=1}^m$ are all n-qubit Pauli strings. By preparing two-copy states $\rho(\boldsymbol{\theta}) \otimes \rho(\boldsymbol{\theta})$ and performing simultaneous Bell measurements between pairs of qubits of these two copies, $M = \mathcal{O}\left(\frac{1}{\varepsilon^2}\right)$ such measurements give a $\omega(\boldsymbol{\theta})$ rich enough to compute any $\mathrm{Tr}[\rho(\boldsymbol{\theta})P_i]^2$ to precision ε [3]. Therefore, evaluating $f_{\boldsymbol{\theta}}(\boldsymbol{x})$ requires only $M \in \widetilde{\mathcal{O}}\left(\frac{\max_{\boldsymbol{x}} \|w(\boldsymbol{x})\|_1^2}{\varepsilon^2}\right)$ measurements using this shadow model, compared to $2^{\Omega(n)}$ for a shadow model that would construct $\omega(\boldsymbol{\theta})$ using single-copy measurements only (assuming that for all $i \in \{1, \ldots, m\}$, there exists an $\boldsymbol{x} \in \mathbb{R}^d$ such that $w(\boldsymbol{x})_i \neq 0$). For a $w(\boldsymbol{x})$ that is k-sparse for all $\boldsymbol{x}$, with $k \ll m$, this constitutes an exponential separation.

C. Complexity classes

Definition 1.4 (BQP). *A language L is in BQP if and only if there exists a polynomial-time uniform family of quantum circuits $\{U_n : n \in \mathbb{N}\}$, such that*

1. *For all $n \in \mathbb{N}$, U_n takes as input an n-qubit computational basis state, and outputs one bit obtained by measuring the first qubit in the computational basis.*
2. *For all $x \in L$, the probability that the output of $U_{|x|}$ applied on the input x is 1 is greater or equal to $2/3$.*
3. *For all $x \notin L$, the probability that the output of $U_{|x|}$ applied on the input x is 0 is greater or equal to $2/3$.*

Definition 1.5 (P/poly). *A language L is in P/poly if and only if there exists a polynomial-time classical algorithm $\mathcal{A}$ and a sequence of polynomial-size advice strings $\{\alpha_n \in \{0,1\}^{poly(n)}\}_{n\in\mathbb{N}}$ such that for all $n \in \mathbb{N}$ and all $x \in \{0,1\}^n$:*

$$\mathcal{A}(x, \alpha_n) = 1 \iff x \in L. \tag{4}$$

Definition 1.6 (BPP/qgenpoly). *A language L is in BPP/qgenpoly if and only if there exists a polynomial-time uniform family of quantum circuits $\{U_n : n \in \mathbb{N}\}$ and a polynomial-time probabilistic classical algorithm $\mathcal{A}$, such that*

1. *For all $n \in \mathbb{N}$, U_n takes as input the computational basis state $|0\rangle^{\otimes m}$ for $m \in \mathcal{O}(poly(n))$, and outputs m bits obtained by measuring all qubits in the computational basis. This constitutes the* quantum-generated *advice ω_n.*
2. *For all $x \in \{0,1\}^*$, $\mathcal{A}$ takes as input x and $\omega_{|x|}$.*
3. *For all $x \in L$, the probability that $\mathcal{A}(x, \omega_{|x|})$ outputs 1 is greater or equal to $2/3$, taken over the randomness of $\omega_{|x|}$ and the internal randomness of $\mathcal{A}$.*
4. *For all $x \notin L$, the probability that $\mathcal{A}(x, \omega_{|x|})$ outputs 0 is greater or equal to $2/3$, taken over the randomness of $\omega_{|x|}$ and the internal randomness of $\mathcal{A}$.*

The following inclusions are easy to show:

$$\mathsf{BPP} \overset{(i)}{\subseteq} \mathsf{BPP/qgenpoly} \overset{(ii)}{\subseteq} \mathsf{BQP}. \tag{5}$$

(i) follows from making the quantum-generated advice ω_n empty in the definition of BPP/qgenpoly. (ii) follows from the ability to efficiently simulate classical computations on a quantum computer. As illustrated in Fig. 2, the

algorithm $\mathcal{A}$ in the definition of $\mathsf{BPP/qgenpoly}$ can be simulated unitarily, and absorbed in the uniform family of quantum circuits $\{U_n : n \in \mathbb{N}\}$, resulting in polynomial-time quantum circuits that fit the definition of BQP.

Our learning separations results, i.e., Theorem 1 in the main text (Lemmas 3.6 and 3.7 in the Appendix) and Theorem 5 in the main text (Lemma 4.2 in the Appendix), can be seen as evidence that the inclusions (i) and (ii) are strict, based on complexity-theory assumptions. Other notable inclusions that are useful to prove our results are:

$$\mathsf{BPP/qgenpoly} \subsetneq \mathsf{BPP/poly} = \mathsf{P/poly}. \tag{6}$$

The equality $\mathsf{BPP/poly} = \mathsf{P/poly}$ follows from the derandomization results of Adleman [4], which show that the random errors made by an algorithm in $\mathsf{BPP/poly}$ can be canceled by an appropriate choice of the random bits used by the randomized algorithm, which are then appended to the poly-sized advice to obtain an algorithm in $\mathsf{P/poly}$. The inclusion $\mathsf{BPP/qgenpoly} \subseteq \mathsf{BPP/poly}$ simply comes from the fact that $\mathsf{BPP/poly}$ is not restricted in *how* the poly-sized advice is generated, and the remainder of its definition is identical to that of $\mathsf{BPP/qgenpoly}$. The restriction we make on *how* the advice is generated in $\mathsf{BPP/qgenpoly}$ makes it a physically relevant complexity class, as opposed to $\mathsf{(BP)P/poly}$. All problems in $\mathsf{BPP/qgenpoly}$ can be solved efficiently (i.e., in polynomial time) using (classical and) quantum computers, while $\mathsf{P/poly}$ notably contains undecidable problems. Consider for instance the unary version of the halting problem: $\text{UHALT} = \{1^n, \text{where } n \text{ encodes } (M, x) \text{ such that the Turing machine } M \text{ halts on } x\}$ is an undecidable language (as any algorithm that would decide it would also be able to decide the traditional halting problem), but by considering the advice $\alpha_n = 1$ if $1^n \in \text{UHALT}$ and 0 otherwise (uniquely defined for each input size n), one trivially obtains an algorithm in $\mathsf{P/poly}$ that solves it. The fact that this advice cannot be generated by a uniform family of (poly-time) circuits is irrelevant for the class $\mathsf{P/poly}$, which is at the source of this result. However it is relevant for the class $\mathsf{BPP/qgenpoly}$, and, in fact, having $\text{UHALT} \in \mathsf{BPP/qgenpoly}$ would mean that one could solve an undecidable problem using uniform (poly-time) circuits. The impossibility of this hypothetical result gives $\mathsf{BPP/qgenpoly} \subsetneq \mathsf{P/poly}$.

2. Properties of flipped models

A. Sample complexity of evaluating quantum models

Consider a linear quantum model (either conventional or flipped) of the form

$$f_{\boldsymbol{y}}(\boldsymbol{x}) = \mathrm{Tr}[\rho(\boldsymbol{x})O(\boldsymbol{y})], \tag{7}$$

for a quantum state $\rho(\boldsymbol{x})$ parametrized by a vector $\boldsymbol{x} \in \mathbb{R}^d$ and $O(\boldsymbol{y})$ a Hermitian observable parametrized by a vector $\boldsymbol{y} \in \mathbb{R}^p$. Assume that we can prepare single copies of $\rho(\boldsymbol{x})$ and that we can measure them in the eigenbasis of $O(\boldsymbol{y})$. We ask: given error parameters $\varepsilon, \delta > 0$, how many such measurements of $\rho(\boldsymbol{x})$ do we need in order to compute an estimate $\widehat{f}_{\boldsymbol{y}}(\boldsymbol{x})$ of $f_{\boldsymbol{y}}(\boldsymbol{x})$ such that $\left|\widehat{f}_{\boldsymbol{y}}(\boldsymbol{x}) - f_{\boldsymbol{y}}(\boldsymbol{x})\right| \leq \varepsilon$ with success probability at least $1-\delta$.

It is easy to see that this problem corresponds to a simple Monte Carlo mean estimation. Indeed, we can write a decomposition of $O(\boldsymbol{y})$ in its eigenbasis as:

$$O(\boldsymbol{y}) = \sum_i \lambda_i(\boldsymbol{y}) \left|\phi_i\right\rangle\!\left\langle\phi_i\right| \tag{8}$$

where $\lambda_i(\boldsymbol{y})$ is a real eigenvalue (since $O(\boldsymbol{y})$ is Hermitian) associated to the eigenstate $\left|\phi_i\right\rangle\!\left\langle\phi_i\right|$ (these eigenstates can in general also depend on $\boldsymbol{y}$, but we do not write this dependence explicitly for ease of notation). We can also write a decomposition of $\rho(\boldsymbol{x})$ in this same basis as :

$$\rho(\boldsymbol{x}) = \sum_{i,j} \rho_{i,j}(\boldsymbol{x}) \left|\phi_i\right\rangle\!\left\langle\phi_j\right| \tag{9}$$

such that $\mathrm{Tr}[\rho(\boldsymbol{x})] = \sum_i \rho_{i,i}(\boldsymbol{x}) = 1$ by the unit-trace property of $\rho(\boldsymbol{x})$, and $\left\langle\phi_i\right| \rho(\boldsymbol{x}) \left|\phi_i\right\rangle = \rho_{i,i}(\boldsymbol{x}) \geq 0$ from its positive semi-definiteness. From these two properties, we deduce that $\{\rho_{i,i}(\boldsymbol{x})\}_i$ defines a probability distribution over the eigenstates $\{\left|\phi_i\right\rangle\!\left\langle\phi_i\right|\}_i$. Therefore, we can see that:

$$\mathrm{Tr}[\rho(\boldsymbol{x})O(\boldsymbol{y})] = \mathrm{Tr}\left[\sum_{i,j,k} \rho_{i,j}(\boldsymbol{x})\lambda_k(\boldsymbol{y}) \left|\phi_i\right\rangle\!\left\langle\phi_j|\phi_k\right\rangle\!\left\langle\phi_k\right|\right] \tag{10}$$

$$= \text{Tr}\left[\sum_{i,j} \rho_{i,j}(\boldsymbol{x})\lambda_j(\boldsymbol{y}) |\phi_i\rangle\langle\phi_j|\right] \tag{11}$$

$$= \sum_i \rho_{i,i}(\boldsymbol{x})\lambda_i(\boldsymbol{y}) \tag{12}$$

simply corresponds to the expectation value of the random variable $i \mapsto \lambda_i(\boldsymbol{y})$ under the probability distribution $\{\rho_{i,i}(\boldsymbol{x})\}_i$, i.e., the probability distribution obtained by measuring $\rho(\boldsymbol{x})$ in the eigenbasis of $O(\boldsymbol{y})$.

Therefore, we can use known results from (classical) Monte Carlo estimation to bound the sample complexity of evaluating this mean value, and therefore the quantum model. With the assumption that $\rho(\boldsymbol{x})$ is given as a black-box and that it can generate arbitrary quantum states (and therefore arbitrary distributions $\{\rho_{i,i}(\boldsymbol{x})\}_i$), the only property of the random variable $i \mapsto \lambda_i(\boldsymbol{y})$ we can use to bound the sample complexity is its bounded domain. Indeed, without additional assumptions of the distribution, we have a tight sample complexity bound of

$$\Theta\left(\frac{B^2 \log(\delta^{-1})}{\varepsilon^2}\right) \tag{13}$$

samples in order to estimate the mean of a random variable taking values in $[-B, B]$, to precision ε and with probability of success $1 - \delta$ [5, 6]. In the case of a quantum model, the random $i \mapsto \lambda_i(\boldsymbol{y})$ takes values in $[-\|O(\boldsymbol{y})\|_\infty, \|O(\boldsymbol{y})\|_\infty]$, where $\|O(\boldsymbol{y})\|_\infty$ is the spectral norm of the observable $O(\boldsymbol{y})$. Therefore, the sample complexity of estimating a quantum model $f_{\boldsymbol{y}}(\boldsymbol{x}) = \text{Tr}[\rho(\boldsymbol{x})O(\boldsymbol{y})]$ is in

$$\Theta\left(\frac{\|O(\boldsymbol{y})\|_\infty^2 \log(\delta^{-1})}{\varepsilon^2}\right), \tag{14}$$

in the absence of any constraint (or information) on the quantum states $\rho(\boldsymbol{x})$.

B. Generalization performance

In this section, we study the generalization performance of flipped models. Our result can be stated informally in the following lemma:

Lemma 2.1 (Generalization bounds (informal)). *Consider a flipped model $f_{\boldsymbol{\theta}}$ that acts on n qubits and has a bounded observable norm $\|O\|_\infty$. If this model achieves a small training error $|f_{\boldsymbol{\theta}}(\boldsymbol{x}) - f(\boldsymbol{x})| \leq \eta$ for all $\boldsymbol{x}$ in a dataset of size M, then it also has a small expected error $|f_{\boldsymbol{\theta}}(\boldsymbol{x}) - f(\boldsymbol{x})| \leq 2\eta$ with probability $1 - \varepsilon$ over the data distribution, provided that the size of the dataset scales as $M \geq \widetilde{\Omega}\left(\frac{n\|O\|_\infty^2}{\varepsilon\eta^2}\right)$.*

To prove this result, we take an approach very similar to that of Aaronson [7], where we lower bound the number of qubits n and spectral norm $\|O\|_\infty$ needed by a flipped model to encode arbitrary k-bit strings, in a way that can be recovered efficiently via repeated measurements. These bounds naturally allow us to upper bound the fat-shattering dimension of flipped models, a complexity measure that is widely used in generalization bounds [8].

1. *Encoding bit-strings in flipped models*

Theorem 2.2. *Let k and n be positive integers with $k > n$. For all k-bit strings $\boldsymbol{y} = y_1 \dots y_k$, let $\rho(\boldsymbol{y})$ be an n-qubit mixed state that "encodes" $\boldsymbol{y}$, meaning each bitstring $\boldsymbol{y}$ is associated to an arbitrary n-qubit quantum state $\rho(\boldsymbol{y})$. Suppose there exist Hermitian observables $O_1, \dots, O_k$ with spectral norms $\|O_i\|_\infty$ such that we call $\|O\|_\infty = \max_i \|O_i\|_\infty$, as well as real numbers $\alpha_1, \dots, \alpha_k$, such that, for all $\boldsymbol{y} \in \{0,1\}^k$ and $i \in \{1, \dots, k\}$,*

(i) if $y_i = 0$, then $\text{Tr}[\rho(\boldsymbol{y})O_i] \leq \alpha_i - \gamma$, and

(ii) if $y_i = 1$, then $\text{Tr}[\rho(\boldsymbol{y})O_i] \geq \alpha_i + \gamma$.

Then $n\|O\|_\infty^2/\gamma^2 \in \Omega(k)$.

Proof. We take a similar approach to the proof of Aaronson [7], in which we show that a combination of an encoding $\rho(\boldsymbol{y})$ and observables $O_1, \dots, O_k$ that satisfies guarantees (i) and (ii) would need $n\|O\|_\infty^2/\gamma^2$ to scale linearly in the

length k of the bit-strings it encodes in order not to contradict with Holevo's bound.

Suppose by contradiction that such an encoding scheme exists with $n\|O\|_\infty^2/\gamma^2 \in o(k)$. We first adapt to the setting of Aaronson by constructing two-outcome POVMs $\{E_i, I - E_i\}$ out of the observables O_i. That is, we take the general Hermitian matrices O_i with eigenvalues in $[\lambda_{\min}, \lambda_{\max}] \subset [-\|O\|_\infty, \|O\|_\infty]$, and transform them into Hermitian matrices E_i with eigenvalues in $[0, 1]$, such that the POVM $\{E_i, I - E_i\}$ accepts ρ (i.e., outputs 1) with probability $\mathrm{Tr}(\rho E_i)$, and rejects ρ (i.e., outputs 0) with probability $1 - \mathrm{Tr}(\rho E_i)$. Specifically, we define

$$E_i = \frac{O_i + |\lambda_{\min}| I}{|\lambda_{\min}| + |\lambda_{\max}|}. \tag{15}$$

Conditions (i) and (ii) then translate to:

$$\begin{cases} (i') \text{ if } y_i = 0, \text{ then } \mathrm{Tr}[\rho(\boldsymbol{y}) E_i] \leq \frac{\alpha_i + |\lambda_{\min}|}{|\lambda_{\min}| + |\lambda_{\max}|} - \frac{\gamma}{|\lambda_{\min}| + |\lambda_{\max}|} \\ (ii') \text{ if } y_i = 1, \text{ then } \mathrm{Tr}[\rho(\boldsymbol{y}) E_i] \geq \frac{\alpha_i + |\lambda_{\min}|}{|\lambda_{\min}| + |\lambda_{\max}|} + \frac{\gamma}{|\lambda_{\min}| + |\lambda_{\max}|} \end{cases} \tag{16}$$

From here, by noting that $|\lambda_{\min}| + |\lambda_{\max}| \leq 2\|O\|_\infty$, we can directly apply Theorem 2.6 of Aaronson [7] and get our result, but we detail the reasoning further for clarity.

We first need to amplify the probability that we correctly identify whether $y_i = 0$ or 1 from measuring copies of $\rho(\boldsymbol{y})$, since the probabilities obtained from E_i can be arbitrarily small. Consider an amplified scheme, where each bit-string $\boldsymbol{y} \in \{0,1\}^k$ is encoded by the tensor product $\rho(\boldsymbol{y})^{\otimes \ell}$, for some $\ell > 1$ to be defined later. For all $i \in \{1, \ldots, k\}$, let $\{E_i^*, I - E_i^*\}$ be the amplified POVM that applies $\{E_i, I - E_i\}$ to each of the ℓ copies of $\rho(\boldsymbol{y})$ and accepts if and only if at least $\widetilde{\alpha}_i \ell = \frac{\alpha_i + |\lambda_{\min}|}{|\lambda_{\min}| + |\lambda_{\max}|}\ell$ of these POVMs do. For all $j \in \{1, \ldots, \ell\}$, call $X_i^{(j)}$ the random variable that takes the value 1 if $\{E_i, I - E_i\}$ accepts the j-th copy of $\rho(\boldsymbol{y})$ (i.e., with probability $p_i = \mathrm{Tr}(\rho(\boldsymbol{y}) E_i)$), and value 0 otherwise.

Consider the case where $y_i = 0$. We have $\mathrm{Tr}[O_i \rho(y)] \leq \alpha_i - \gamma$, which implies that $\widetilde{\alpha}_i \geq p_i + \frac{\gamma}{|\lambda_{\min}| + |\lambda_{\max}|}$. Therefore, the probability that at least $\widetilde{\alpha}_i \ell$ of the POVMs accept is then:

$$P(\overline{X}_i \geq \widetilde{\alpha}_i) \leq P\left(\overline{X}_i \geq p_i + \frac{\gamma}{|\lambda_{\min}| + |\lambda_{\max}|}\right) \tag{17}$$

for $\overline{X}_i = \frac{1}{\ell}\sum_{j=1}^{\ell} X_i^{(j)}$. From the Chernoff bound, we hence get:

$$P(\overline{X}_i \geq \widetilde{\alpha}_i) \leq e^{-2\left(\frac{\gamma}{|\lambda_{\min}| + |\lambda_{\max}|}\right)^2 l}. \tag{18}$$

To guarantee an acceptance probability $\mathrm{Tr}(\rho(\boldsymbol{y})^{\otimes \ell} E_i^*) \leq 1/3$, it is then sufficient to take $\ell = \left\lceil \frac{2\log(3)\|O\|_\infty^2}{\gamma^2} \right\rceil$. A similar analysis holds for the case $y_i = 1$.

From here, the result we use that derives from Holevo's bound is Theorem 5.1 of Ambainis *et al.* [9]. It states that in order for the POVMs $\{E_i^*, I - E_i^*\}$ to correctly identify whether $y_i = 0$ or 1 with probability of failure less than 1/3, we need a number of qubits $n\ell \geq (1 - H(1/3))k$, where H is the binary entropy function. This implies that $n\|O\|_\infty^2/\gamma^2 \geq \frac{2(1-H(1/3))}{\log(3)} k \in \Omega(k)$. □

2. *Generalization bounds of flipped models*

The conditions (i) and (ii) of Theorem 2.2 are very similar to that of a fat-shattering dimension of a concept class.

Definition 2.3. *Let $\mathcal{X}$ be a data space, let $\mathcal{C}$ be a class of functions from $\mathcal{X}$ to $\mathbb{R}$, and let $\gamma > 0$. The fat-shattering dimension of the concept class $\mathcal{C}$ at width γ, denoted $\mathrm{fat}_{\mathcal{C}}(\gamma)$, is defined as the size k of the largest set of points $\{\boldsymbol{x}^{(1)}, \ldots, \boldsymbol{x}^{(k)}\}$ for which there exist real numbers $\alpha_1, \ldots, \alpha_k$ such that for all $\boldsymbol{y} \in \{0,1\}^k$, there exists a $f \in \mathcal{C}$ that satisfies, for all $i \in \{1, \ldots, k\}$,*

(i) if $y_i = 0$, then $f(\boldsymbol{x}) \leq \alpha_i - \gamma$, and

(ii) if $y_i = 1$, then $f(\boldsymbol{x}) \geq \alpha_i + \gamma$.

By comparing this definition with the statement of Theorem 2.2, we can show:

Corollary 2.4. *Consider a flipped model $f_{\boldsymbol{\theta}}(\boldsymbol{x}) = \mathrm{Tr}[\rho(\boldsymbol{\theta})O(\boldsymbol{x})]$ defined on a data space $\mathcal{X}$, using n-qubit quantum states and observables with spectral norm $\|O\|_\infty = \sup_{\boldsymbol{x}\in\mathcal{X}} \|O(\boldsymbol{x})\|_\infty$. Call $\mathcal{C}_{n,O} = \{f_{\boldsymbol{\theta}}\}_{\boldsymbol{\theta}}$ the concept (or hypothesis) class associated to this model. Then, for all $\gamma > 0$, we have $\mathrm{fat}_{\mathcal{C}_{n,O}}(\gamma) \in \mathcal{O}(n\|O\|_\infty^2/\gamma^2)$.*

Proof. We note that since, for all $\boldsymbol{x} \in \mathcal{X}$, $O(\boldsymbol{x})$ lives in a manifold of all observables with spectral norm $\|O\|_\infty$, then the fat-shattering dimension of $\mathcal{C}_{n,O}$ is upper bounded by that of the concept class $\widetilde{\mathcal{C}}_{n,O} = \{O' \mapsto \mathrm{Tr}[\rho(\boldsymbol{\theta})O']\}_{\boldsymbol{\theta}}$ defined on the input space of observables O' that satisfy $\|O'\|_\infty \leq \|O\|_\infty$. Theorem 2.2 immediately yields an upper bound for $\mathrm{fat}_{\widetilde{\mathcal{C}}_{n,O}}(\gamma)$, when we identify $\rho(\boldsymbol{\theta})$ in this corollary to $\rho(\boldsymbol{y})$ in the Theorem, and observe that the number of observables $O_1, \dots, O_k$ that can be γ-shattered (i.e., conditions (i) and (ii) for all labelings) must satisfy $k \in \mathcal{O}(n\|O\|_\infty^2/\gamma^2)$. □

To obtain generalization bounds on the performance of flipped models, we combine this bound on their fat-shattering dimension with standard results from learning theory, e.g.:

Theorem 2.5 (Anthony and Barlett [8])**.** *Let $\mathcal{X}$ be a data space, let $\mathcal{C}$ be a class of functions from $\mathcal{X}$ to $\mathbb{R}$, and let $\mathcal{D}$ be a probability measure over $\mathcal{X}$. Fix an element $f \in \mathbb{R}^{\mathcal{X}}$, as well as error parameters $\varepsilon, \eta, \gamma, \delta > 0$ with $\gamma > \eta$. Suppose that we draw m samples $X = (\boldsymbol{x}^{(1)}, \dots, \boldsymbol{x}^{(m)})$ from $\mathcal{X}$ according to $\mathcal{D}$, and choose any hypothesis $h \in \mathcal{C}$ such that $|h(\boldsymbol{x}) - f(\boldsymbol{x})| \leq \eta$ for all $\boldsymbol{x} \in X$. Then, there exists a positive constant K such that, provided*

$$m \geq \frac{K}{\varepsilon}\left(\mathrm{fat}_{\mathcal{C}}\left(\frac{\gamma-\eta}{8}\right)\log^2\left(\frac{\mathrm{fat}_{\mathcal{C}}\left(\frac{\gamma-\eta}{8}\right)}{(\gamma-\eta)\varepsilon}\right) + \log\left(\frac{1}{\delta}\right)\right),$$

with probability $1-\delta$,

$$\mathrm{Pr}_{\boldsymbol{x}\in\mathcal{D}}[|h(\boldsymbol{x}) - f(\boldsymbol{x})| > \gamma] \leq \varepsilon.$$

From Corollary 2.4, we have that

$$m \in \widetilde{\Omega}\left(\frac{1}{\varepsilon}\left(\frac{n\|O\|_\infty^2}{(\gamma-\eta)^2} + \log\left(\frac{1}{\delta}\right)\right)\right)$$

samples suffice to get these generalization guarantees. This proves Lemma 2.1.

C. Flipping bounds

In this section, we study mappings between conventional and flipped models (most importantly from conventional to flipped, but our flipping bounds can be used either way). We find that the important quantity that governs the trade-off in resources between these models is the observable trace norm $\|O\|_1 = \mathrm{Tr}\left[\sqrt{O^2}\right]$ of the model to be mapped. Since all observables $O(\boldsymbol{y})$ (where $\boldsymbol{y}$ is either a data vector or a parameters vector, depending on the model) have to be turned into unit-trace density matrices, their eigenvalues need (i) either to be normalized when preserving the number of qubits of the model, or (ii) be encoded in more qubits than in the original model (e.g., by using a binary encoding of all the eigenvalues of $O(\boldsymbol{y})$). Each of these options has its disadvantages: (i) normalizing eigenvalues introduces an overhead in the spectral norm $\|O'\|_\infty$ of the observable of the resulting model, which results in an overhead in the number of measurements needed to evaluate this model to the same precision as the original one. As for (ii), we show that the number of qubits of the new model would need to scale quadratically with $\|O\|_1$ in general, which is commonly an exponential quantity in the number of qubits of the original model (e.g., when O is a Pauli). We show the following lemma:

Lemma 2.6 (Flipping bounds (informal))**.** *Any conventional linear model $f_{\boldsymbol{\theta}}(\boldsymbol{x}) = \mathrm{Tr}[\rho(\boldsymbol{x})O(\boldsymbol{\theta})]$ acting on n qubits and with a bounded observable trace norm $\|O\|_1 \leq d$ admits an equivalent flipped model $\widetilde{f}_{\boldsymbol{\theta}}(\boldsymbol{x}) = \mathrm{Tr}[\rho'(\boldsymbol{\theta})O'(\boldsymbol{x})]$ acting on $m = n+1$ qubits and with observable spectral norm $\|O'\|_\infty = d$. The bound on the spectral norm is essentially tight in the regime where $n, m \in \mathcal{O}(\log(d))$, i.e., in this case we have $\|O'\|_\infty \geq \widetilde{\Omega}(d)$.*

Theorem 2.7. *Given a specification of a conventional quantum model $f_{\boldsymbol{\theta}}(\boldsymbol{x}) = \mathrm{Tr}[\rho(\boldsymbol{x})O(\boldsymbol{\theta})]$ acting on n qubits, with a known (upper bound on the) trace norm $\|O\|_1$ of its observable, one can construct an equivalent flipped model $\widetilde{f}_{\boldsymbol{\theta}}(\boldsymbol{x}) = \mathrm{Tr}[\rho'(\boldsymbol{\theta})O'(\boldsymbol{x})]$ acting on $n+1$ qubits such that $\widetilde{f}_{\boldsymbol{\theta}}(\boldsymbol{x}) = f_{\boldsymbol{\theta}}(\boldsymbol{x})$, $\forall \boldsymbol{x}, \boldsymbol{\theta}$ and $\|O'\|_\infty = \|O\|_1$.*

Proof. From Def. 1.1, we assume that, in the definition of the conventional model, $\rho(\boldsymbol{x})$ is obtained by applying a unitary $U(\boldsymbol{x})$ on a known quantum state ρ_0 that is diagonal in the computational basis (i.e., is a mixture of computational basis states). As for $O(\boldsymbol{\theta})$, we assume that it is specified by a unitary $V(\boldsymbol{\theta})$ and a weighted sum of d Hermitian operators O_i, such that $O(\boldsymbol{\theta}) = \sum_{i=1}^d w_i V(\boldsymbol{\theta}) O_i V^\dagger(\boldsymbol{\theta})$ and for each $i \in \{1, \dots, d\}$ we know how to decompose O_i as $O_i = \sum_{j=0}^{2^n-1} \lambda_{i,j} W_i |j\rangle\langle j| W_i^\dagger$, for some known $\lambda_{i,j}$'s and W_i's. Note that, as opposed to the parameters that specify $V(\boldsymbol{\theta})$, the weights w_i influence the trace norm $\|O(\boldsymbol{\theta})\|_1$. Therefore we need to pay attention to the fact that $\|O(\boldsymbol{\theta})\|_1$ is only upper bounded by $\|O\|_1 = \sup_{\boldsymbol{\theta}} \|O(\boldsymbol{\theta})\|_1$, and that these two quantities are not always equal.

Algorithm 1: Flipped evaluation of a conventional model

Input: an n-qubit unitary $U(\boldsymbol{x})$ and a quantum state ρ_0 (diagonal in the computation basis) such that $\rho(\boldsymbol{x}) = U(\boldsymbol{x})\rho_0 U^\dagger(\boldsymbol{x})$, n-qubit unitaries $V(\boldsymbol{\theta})$ and $\{W_i\}_{1\leq i\leq d}$, real values $\{w_i\}_{i=1}^d$, $\{\lambda_{i,j}\}_{0\leq j\leq 2^n-1}^{1\leq i\leq d}$, such that $O(\boldsymbol{\theta}) = \sum_i w_i V(\boldsymbol{\theta}) O_i V^\dagger(\boldsymbol{\theta})$ with $O_i = \sum_j \lambda_{i,j} W_i |j\rangle\langle j| W_i^\dagger$.
Output: A flipped evaluation of the conventional model $\mathrm{Tr}[\rho(\boldsymbol{x})O(\boldsymbol{\theta})]$

1 Initialize $o = 0$, $N = \mathcal{O}(\|O\|_1^2/\varepsilon^2)$;
2 **for** N *iterations* **do**
3 Sample $i \in \{1, \dots, d+1\}$ w.p. $\left(\frac{w_1\|O_1\|_1}{\|O\|_1}, \dots, \frac{w_d\|O_d\|_1}{\|O\|_1}, \frac{\|O\|_1 - \sum_{i=1d} w_i \|O_i\|_1}{\|O\|_1}\right)$;
4 **if** $i = d+1$ **then**
5 break iteration (or alternatively prepare $\sigma(\boldsymbol{\theta}) = I/2^{n+1}$ and jump to line 9);
6 Sample $b \in \{+,-\}$ w.p. $\frac{\|O_{i,b}\|_1}{\|O_i\|_1}$;
7 Sample $j \in \{0, \dots, 2^n-1\}$ w.p. $\frac{\max(0, b\lambda_{i,j})}{\|O_{i,b}\|_1}$;
8 Prepare $\sigma(\boldsymbol{\theta}) = |\widetilde{b}\rangle\langle\widetilde{b}| \otimes V(\boldsymbol{\theta}) W_i |j\rangle\langle j| W_i^\dagger V^\dagger(\boldsymbol{\theta})$, for $\widetilde{b} = 2b-1$;
9 Measure $(I \otimes U^\dagger(\boldsymbol{x}))\sigma(\boldsymbol{\theta})(I \otimes U(\boldsymbol{x}))$ in the computational basis and call the outcome $|\widetilde{b}\rangle \otimes |j\rangle$;
10 $o \leftarrow o + b\|O\|_1 \rho_{0,j}$, where $\rho_{0,j}$ is the j-th diagonal element of ρ_0;
11 **return** o/N

Out of the observables $O(\boldsymbol{\theta})$, we need to prepare quantum states $\rho'(\boldsymbol{\theta})$ such that $\mathrm{Tr}[\rho'(\boldsymbol{\theta})O'(\boldsymbol{x})] = \mathrm{Tr}[\rho(\boldsymbol{x})O(\boldsymbol{\theta})]$. The only difficulty is that quantum states are positive semi-definite and have unit trace while Hermitian observables generally do not fulfill any of these two conditions. To get around these constraints, we simply decompose the observables $O(\boldsymbol{\theta})$ into positive and negative components, that we both normalize. More precisely, call $O_+(\boldsymbol{\theta})$ ($O_-(\boldsymbol{\theta})$) the positive (negative) part of $O(\boldsymbol{\theta}) = O_+(\boldsymbol{\theta}) - O_-(\boldsymbol{\theta})$. We define:

$$\begin{cases} \rho'_+(\boldsymbol{\theta}) = O_+(\boldsymbol{\theta})/\|O_+(\boldsymbol{\theta})\|_1 \\ \rho'_-(\boldsymbol{\theta}) = O_-(\boldsymbol{\theta})/\|O_-(\boldsymbol{\theta})\|_1 \end{cases} \quad \text{and} \quad \begin{cases} p_+ = \|O_+(\boldsymbol{\theta})\|_1/\|O(\boldsymbol{\theta})\|_1 \\ p_- = \|O_-(\boldsymbol{\theta})\|_1/\|O(\boldsymbol{\theta})\|_1 \end{cases} \tag{19}$$

such that

$$\rho'(\boldsymbol{\theta}) = p_+ |0\rangle\langle 0| \otimes \rho'_+(\boldsymbol{\theta}) + p_- |1\rangle\langle 1| \otimes \rho'_-(\boldsymbol{\theta}) \tag{20}$$

is a valid quantum state (positive semi-definite and unit trace). We can then take

$$O'(\boldsymbol{x}) = \|O(\boldsymbol{\theta})\|_1 (|0\rangle\langle 0| - |1\rangle\langle 1|) \otimes \rho(\boldsymbol{x}) \tag{21}$$

which, as one can easily verify, leads to $\mathrm{Tr}[\rho'(\boldsymbol{\theta})O'(\boldsymbol{x})] = \mathrm{Tr}[\rho(\boldsymbol{x})O(\boldsymbol{\theta})]$.
However, this still does not give us a proper flipped model as the renormalization factor $\|O(\boldsymbol{\theta})\|_1$ of $O'(\boldsymbol{x})$ can depend on the parameters $\boldsymbol{\theta}$ (and more precisely the weights w_i, see remark above). We would like to use here the upper bound $\|O\|_1$. To do so, we can simply (re-)define:

$$\begin{cases} p_+ = \|O_+(\boldsymbol{\theta})\|_1/\|O\|_1 \\ p_- = \|O_-(\boldsymbol{\theta})\|_1/\|O\|_1 \end{cases} \quad \text{and} \quad p_0 = \frac{\|O\|_1 - \|O_+(\boldsymbol{\theta})\|_1 - \|O_-(\boldsymbol{\theta})\|_1}{\|O\|_1} \tag{22}$$

and also

$$\begin{cases} \rho'(\boldsymbol{\theta}) = p_+ \,|0\rangle\langle 0| \otimes \rho'_+(\boldsymbol{\theta}) + p_- \,|1\rangle\langle 1| \otimes \rho'_-(\boldsymbol{\theta}) + p_0 I/2^{n+1} \\ O'(\boldsymbol{x}) = \|O\|_1 (|0\rangle\langle 0| - |1\rangle\langle 1|) \otimes \rho(\boldsymbol{x}) \end{cases} \tag{23}$$

such that we still have $\mathrm{Tr}[\rho'(\boldsymbol{\theta})O'(\boldsymbol{x})] = \mathrm{Tr}[\rho(\boldsymbol{x})O(\boldsymbol{\theta})]$ but where we have now defined a proper flipped model. □

Going a bit further, we also propose an algorithm to evaluate the flipped model we constructed in our proof (see Algorithm 1). Given that we only assume to know the eigenvalue decomposition of the single O_i's, and not that of the full observable $O(\boldsymbol{\theta})$, we do not decompose $O(\boldsymbol{\theta})$ directly into its positive and negative components, but rather the O_i's. For this, we define:

$$\begin{cases} O_{i,+} = \sum_{j,\lambda_{i,j}\geq 0} \lambda_{i,j} W_i \,|j\rangle\langle j|\, W_i^\dagger \\ O_{i,-} = \sum_{j,\lambda_{i,j}\leq 0} |\lambda_{i,j}| W_i \,|j\rangle\langle j|\, W_i^\dagger \end{cases} \tag{24}$$

We then recover $\rho'(\boldsymbol{\theta})$ via importance sampling of the indices i, j and $\pm$ and the implementation of the pure state $V(\boldsymbol{\theta})W_i\,|j\rangle$ (see Algorithm 1)[1]. Naturally, one could alternatively design a full unitary implementation of $\rho'(\boldsymbol{\theta})$ using auxiliary qubits to prepare coherent encodings of the probability distributions appearing in Algorithm 1, along with controlled operations between these auxiliary qubits and the working register, but this would require more qubits and a more complicated quantum implementation.

2. *Lower bounds*

Theorem 2.8. *For d an arbitrary positive integer, there exists a conventional model $\mathrm{Tr}[\rho(\boldsymbol{x})O(\boldsymbol{y})]$, acting on $n \in \mathcal{O}(\log(d))$ qubits, that satisfies $\|O(\boldsymbol{y})\|_1 = d$ for all $\boldsymbol{y} \in \mathcal{Y}$, such that for any flipped model $\mathrm{Tr}[\rho'(\boldsymbol{y})O'(\boldsymbol{x})]$ acting on m qubits with $\|O'\|_\infty = \sup_{\boldsymbol{x}\in\mathcal{X}} \|O'(\boldsymbol{x})\|_\infty$, and any $\varepsilon \geq 0$, if the model satisfies*

$$|\mathrm{Tr}[\rho(\boldsymbol{x})O(\boldsymbol{y})] - \mathrm{Tr}[\rho'(\boldsymbol{y})O'(\boldsymbol{x})]| \leq \varepsilon \quad \forall \boldsymbol{x}, \boldsymbol{y} \in \mathcal{X} \times \mathcal{Y} \tag{25}$$

then, it must also satisfy

$$m\|O'\|_\infty^2 \in \Omega(d^2(1/2 - \varepsilon)^2).$$

Proof. The core of the proof is to show that a conventional model $\mathrm{Tr}[\rho(i)O(\boldsymbol{y})]$ with trace norm $\|O(\boldsymbol{y})\|_1 = d$ and acting on $n = \lceil \log_2(N+1) \rceil$ qubits, for $N = \lfloor d^2/4 \rfloor$, can represent the function $i \mapsto y_i$ for $1 \leq i \leq N$, for all $\boldsymbol{y} \in \{0,1\}^N$. For this, we take, for all $1 \leq i \leq N$:

$$\rho(i) = \frac{1}{2}(|0\rangle + |i\rangle)(\langle 0| + \langle i|) \quad \text{and} \quad O(\boldsymbol{y}) = \sum_{i'=1}^{N+1} O_{i'}(\boldsymbol{y}) \tag{26}$$

for

$$O_i(\boldsymbol{y}) = \begin{cases} y_i(|0\rangle\langle i| + |i\rangle\langle 0|) & \text{if } 1 \leq i \leq N \\ (d - \sqrt{|\boldsymbol{y}|})\,|N+1\rangle\langle N+1| & \text{if } i = N+1 \end{cases} \tag{27}$$

where $|\boldsymbol{y}| = \sum_{i=1}^N y_i$ is the Hamming weight of $\boldsymbol{y}$. By construction, we have that the upper-left $N \times N$ block of $O(\boldsymbol{y})$ satisfies $\left\|O_{(N\times N)}(\boldsymbol{y})\right\|_1 = \sqrt{|\boldsymbol{y}|} \leq d$ (it corresponds to the adjacency matrix of a star graph of degree $D = |\boldsymbol{y}| \leq N$, which has trace norm $2\sqrt{D}$ [10]), such that $\|O(\boldsymbol{y})\|_1 = d$ for all $\boldsymbol{y} \in \{-1,1\}^N$. Also, it is easy to check that $\mathrm{Tr}[\rho(i)O(\boldsymbol{y})] = y_i$ for all $1 \leq i \leq N$.
We take this conventional model to be our target model. Satisfying the condition of Eq. (25) is then equivalent to satisfying:

$$|\mathrm{Tr}[\rho'(\boldsymbol{y})O'(i)] - y_i| \leq \varepsilon \quad \forall\, i, \boldsymbol{y} \in \{1, \ldots, N\} \times \{0,1\}^N.$$

Now note that this condition is stronger than that of Theorem 2.2 for $\gamma = 1/2 - \varepsilon$ and $\alpha_{i,j} = 1/2\ \forall i,j$. Therefore, in order not to contradict with this theorem, we must have $m\|O'\|_\infty^2 \in \Omega(N(1/2-\varepsilon)^2) = \Omega(d^2(1/2-\varepsilon)^2)$. □

Lemma 2.6 is obtained from Theorem 2.7 as stated above and Theorem 2.8 for the case $m \in \mathcal{O}(\log d)$, such that the statement $m\|O'\|_\infty^2 \in \Omega(d^2(1/2-\varepsilon)^2)$ becomes $\|O'\|_\infty \in \Omega\left(\frac{d}{\sqrt{\log(d)}}(1/2-\varepsilon)\right)$.

---

[1] In Algorithm 1, $\sigma(\boldsymbol{\theta})$ corresponds to either $\rho'_+(\boldsymbol{\theta})$ or $\rho'_-(\boldsymbol{\theta})$, depending on the sampled $b \in \{+,-\}$.

3. *Circumventing lower bounds*

Note that our flipping bounds are essentially tight only in the regime where the number of qubits used by the original and resulting model n, m are both in $\mathcal{O}(\text{polylog}(\|O\|_1))$. This is a relevant regime, as it includes notably the case of Pauli observables (be they local or non-local) or linear combinations thereof. However, outside this regime our bounds can be circumvented.

Also note that an easy way of circumventing our lower bounds, even in the regime where $n, m \in \mathcal{O}(\text{polylog}(\|O\|_1))$, is by imposing the constraint that $O(\boldsymbol{y})$ is parametrized by $|\boldsymbol{y}| = \mathcal{O}(\text{poly}(n))$ parameters[2]. Indeed, in this case, one can simply use a similar construction to that in Ref. [11] (Fig. 3) where one would encode $\boldsymbol{y}$ (e.g., in binary form) in auxiliary qubits as $|\widetilde{\boldsymbol{y}}\rangle$ and use controlled operations that are independent of $\boldsymbol{y}$ to simulate the action of gates parametrized by $\boldsymbol{y}$. One can then note that by taking $\rho'(\boldsymbol{\theta}) = \rho_0 \otimes |\widetilde{\boldsymbol{y}}\rangle\langle\widetilde{\boldsymbol{y}}|$ and the rest of the resulting circuit to define $O'(\boldsymbol{x})$, one ends up with a flipped model that acts on $\mathcal{O}(\text{poly}(n))$ qubits and satisfies $\|O'\|_\infty = \|O\|_\infty$. For O defined by a Pauli observable for instance, we have $\|O\|_\infty = 1$ and $\|O\|_1 = 2^n$. Such a construction therefore does not suffer from an exploding spectral norm $\|O'\|_\infty$. However, it also does not lead to shadowfiable models in general as the parametrized states $\rho'(\boldsymbol{\theta})$ play a trivial role and the observables $O'(\boldsymbol{x})$ hide all of the quantum computation.

3. Quantum advantage using shadow models

A. Discrete cube root learning task

In this section we rigorously define the discrete cube root learning task introduced in the main text and detail the proof of its classical hardness. More precisely, we start by introducing the discrete cube root problem and state formally its classical hardness assumption (the discrete cube root assumption). Then we construct a learning task that is classically hard based on this assumption.

1. *The discrete cube root problem*

A definition of the discrete cube root problem can be found in Ref. [12]. For convenience, we restate it in this appendix.[3] Consider two large prime numbers p and q of the form $3k+2,\ 3k'+2$, for distinct k, k', and which can be represented by approximately the same number of bits. Let $N = pq$ be the product of these primes, which we assume to be an n-bit integer, and let $\mathbb{Z}_N = \{0, \ldots, N-1\}$.

We consider the "discrete cube" function $f_N(y) : \mathbb{Z}_N \to \mathbb{Z}_N$ defined as $f_N(y) = y^3 \bmod N$, as well as its inverse, which we denote as $g_N(x) = f_N^{-1}(x) = \sqrt[3]{x} \bmod N$ (see Supplementary Figure 1). As we explain in the following, f_N is particularly interesting because it is believed to be a *one-way function*: $f_N(y)$ can be computed efficiently classically using modular exponentiation, while $g_N(x)$ is believed hard to compute classically, with only knowledge of N and x (and not the factors p, q of N). But first, let us show that the inverse function g_N is properly defined.

Lemma 3.1. *For p, q two distinct prime numbers of the form $3k+2,\ 3k'+2$ and $N = pq$, the function $f_N(y) = y^3 \bmod N$ is a bijection on $\mathbb{Z}_N$.*

Proof. To show that f_N is a bijection on $\mathbb{Z}_N$, it is sufficient to show that it is injective, since it maps $\mathbb{Z}_N$ to itself.
Consider $y, z \in \mathbb{Z}_N$ such that $f_N(y) = f_N(z)$, i.e., $y^3 \bmod N = z^3 \bmod N$. We then have $y^3 - z^3 \equiv 0 \bmod N$, which means that there exists an $l \in \mathbb{N}$ such that $y^3 - z^3 = lN = lpq$. Therefore, we know that $y^3 - z^3$ is divisible by both p and q, which implies $y^3 \equiv z^3 \bmod p$ and $y^3 \equiv z^3 \bmod q$. From here, we apply a similar reasoning for p and q, that we detail only for p.
Given that we assumed $p = 3k+2$, we know that $\gcd(p-1, 3) = 1$. Therefore, Euclid's algorithm assures that there exist $d_p, d'_p \geq 1$ such that $3d_p = (p-1)d'_p + 1$. Then notice that:

$$y^{3d_p} \equiv y^{(p-1)d'_p+1} \equiv y \bmod p \tag{28}$$

where the last congruence follows from applying Fermat's little theorem ($y^p \equiv y \bmod p$ for all $y \in \mathbb{N}$) to show by induction on $m \geq 0$ that $y^{(p-1)m+1} \equiv y \bmod p$. Similarly, $z^{3d_p} \equiv z \bmod p$, and therefore by raising to the power d_p

---

[2] In our proof of Theorem 2.8, we use $|\boldsymbol{y}| \in \Omega(\|O\|_1^2)$, which is in $\Omega(\exp(n))$ for $n, m \in \mathcal{O}(\log(\|O\|_1))$, and subexponential in n for $n, m \in \mathcal{O}(\text{polylog}(\|O\|_1))$

[3] Note that, as opposed to the exposition of Ref. [12], we consider the domain $\mathbb{Z}_N = \{0, \ldots, N-1\}$ instead of $\{i \mid 0 < i < N,\ \gcd(i, N) = 1\}$ for the functions we define next. This allows us to apply more easily the result of Ref. [13] and construct a learning task with a stronger form of classical hardness.

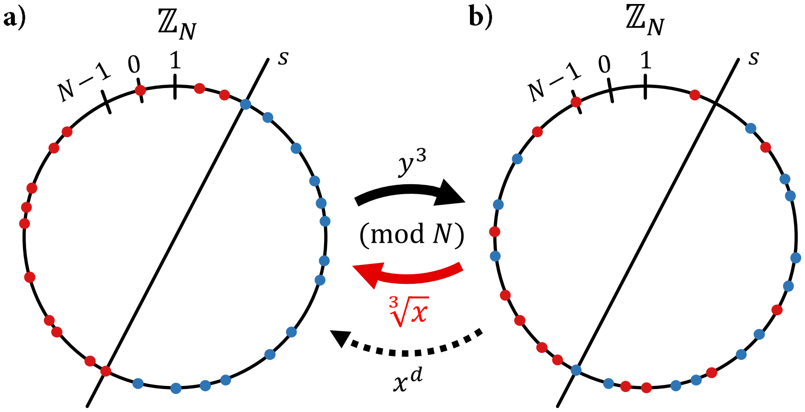

Supplementary Figure 1. **A visualization of the functions involved in the quantum advantage learning task.** The core functions of this task map $\mathbb{Z}_N = \{0, \ldots, N-1\}$ to itself, for N a large semiprime. a) In feature space, data is linearly separable by a hyperplane parametrized by a certain $s \in \mathbb{Z}_N$. One can efficiently transform data y in feature space into its corresponding data x in input space via the "discrete cube" function $x = y^3 \bmod N$. b) To a fully classical learner, data in input space looks randomly labeled, as inverting it back to feature space via the discrete cube root function $y = \sqrt[3]{x} \bmod N$ is believed to be classically intractable. However, a shadow model can make use of the trap-door property of the discrete cube root function to efficiently compute a key $d \in \mathbb{Z}_N$ using a quantum computer and classically map data to feature space through the transformation $y = x^d \bmod N$.

both terms in $y^3 \equiv z^3 \bmod p$, we get $y \equiv z \bmod p$.
From the same reasoning, we get $y \equiv z \bmod q$, which implies that $x - y$ is divisible by both p and q. Since they are distinct primes, then $x - y$ is also divisible by $pq = N$, which means that $x \equiv y \bmod N$. This shows that f_N is injective, and therefore bijective. □

Now that we have shown that the discrete cube root function is properly defined on $\mathbb{Z}_N$, let us define the discrete cube root problem:

Definition 3.2 (Discrete cube root problem)**.** *Let p and q be two distinct primes of the form $3k+2, 3k'+2$ represented by approximately the same number of bits, and such that $N = pq$ is an n-bit integer. Given as input both N and $x \in \mathbb{Z}_N$, output $y \in \mathbb{Z}_N$ such that $y^3 = x \bmod N$.*

The assumption that the "discrete cube" function f_N is a one-way function is formalized by the so-called discrete cube root assumption, which is a special case of the RSA assumption for the exponent $e = 3$.

Definition 3.3 (Discrete cube root assumption [12])**.** *For any polynomial $P(.)$, there does not exist a classical algorithm $\mathcal{A}$, that runs in time $P(n)$ and that, on input N and x (where N is the n-bit product of two random primes of the form $3k+2$ and x is chosen randomly from $\mathbb{Z}_N$), outputs $y \in \mathbb{Z}_N$ such that with probability $1/P(n)$ satisfies $y^3 = x \bmod N$. The probability of success is taken over the random draws of the two primes p, q and the input $x \in \mathbb{Z}_N$ and any internal randomisation of $\mathcal{A}$.*

While other one-way functions like the discrete exponential (along with its inverse, the discrete logarithm) also have similar classical hardness assumptions, the discrete cube (root) function is additionally known to be a *trap-door function*. That is, there exists a key $d \in \mathbb{Z}_N$ such that $g_N(x)$ can be alternatively computed via modular exponentiation as $g_N(x) = x^d \bmod N$. This key d can be efficiently computed from the prime factors p and q of N. We show this using a similar reasoning to that around Eq. (28). Call $\phi(N) = (p-1)(q-1)$. From $p = 3k+2$ and $q = 3k'+2$, we have $\gcd(\phi(N), 3) = 1$. Therefore, Euclid's algorithm assures that there exists $d, d' \geq 1$ such that $3d = \phi(N)d' + 1 = (p-1)(q-1)d' + 1$. We want to show that, for all $y \in \mathbb{Z}_N$,

$$y^{3d} \equiv y \bmod N. \tag{29}$$

To do this, we show that $y^{3d} - y$ is divisible by both p and q. In the case of p, we have:

$$y^{3d} \equiv y^{(p-1)(q-1)d'+1} \equiv y \bmod p. \tag{30}$$

The last equality follows again from Fermat's little theorem (see Eq. (28)). Similarly, $y^{3d} \equiv y \bmod q$, which implies that $y^{3d} - y$ is divisible by p and q, and that $y^{3d} \equiv y \bmod N$. Therefore d is a valid key for computing $g_N(y)$ for all

$y \in \mathbb{Z}_N$. When knowing the factors p and q of N, one can compute $\phi(N)$ and use Euclid's algorithm to find d such that $3d = 1 \bmod \phi(N)$. However, factoring a large N is believed to be computationally intractable classically, which justifies the discrete cube root assumption (Def. 3.3).

2. The learning task

Based on the discrete cube root assumption, we can construct a learning task that is not efficiently PAC learnable classically. In order to define the concept class of this learning task, we first consider the class of functions:

$$\mathcal{F}_n = \{g_N : \mathbb{Z}_N \to \mathbb{Z}_N \mid g_N(x) = \sqrt[3]{x} \bmod N, \text{ for } N \text{ an } n\text{-bit integer that satisfies the DCR conditions}\} \tag{31}$$

defined for any integer n. We define the concept class:

$$\mathcal{C}_n = \{g_{N,s} : \mathbb{Z}_N \to \{0,1\} \mid g_{N,s}(x) = \begin{cases} 1, & \text{if } g_N(x) \in [s, s + \frac{N-1}{2}], \\ 0, & \text{otherwise.} \end{cases}, \text{ for } g_N \in \mathcal{F}_n,\ s \in \mathbb{Z}_N\} \tag{32}$$

for any integer n. In our proofs of classical hardness and quantum learnability, we also need that the integer N corresponding to the target function should be specified with the training data. One way of doing so is to append it to all inputs $x \in \mathbb{Z}_N$ as (x, N) and redefine the concept class accordingly. To ease notation, we assume this transformation to be done implicitly in the following.

Because the discrete cube function is a one-way function and is bijective on $\mathbb{Z}_N$, it is easy to classically generate training data for any concept $g_{N,s} \in \mathcal{C}_n$, under the uniform distribution over $\mathbb{Z}_N$. Indeed, one can simply uniformly sample $y \in \mathbb{Z}_N$, compute its corresponding $x = y^3 \bmod N$ via modular exponentiation and keep from y only the label indicating whether $y \in [s, s + \frac{N-1}{2}]$. The bijectivity of f_N ensures that x is generated uniformly over $\mathbb{Z}_N$.

In the PAC setting, a learning algorithm has to find, for every concept $g_{N,s} \in \mathcal{C}_n$, for every data distribution $\mathcal{D}$, and for all $\varepsilon, \delta \in (0, 1/2)$, a hypothesis function h that, with probability $1 - \delta$, satisfies $\Pr_{x\sim\mathcal{D}}[g_{N,s}(x) \neq h(x)] \leq \varepsilon$ in time and number of samples both polynomial in n, $1/\varepsilon$ and $1/\delta$.

Note that in Ref. [12], the authors consider instead the concept class

$$\mathcal{C}'_n = \{g_{N,i} : \mathbb{Z}_N \to \{0,1\} \mid g_{N,i}(x) = \mathrm{bin}(i, g_N(x)), \text{ for } g_N \in \mathcal{F}_n,\ i \in \{1, \ldots, n\}\} \tag{33}$$

for any integer n, where $\mathrm{bin}(i, y)$ denotes the i-th bit of y in binary form. This concept class is also not efficiently PAC learnable, although this is only shown in a much weaker sense. The authors show that no classical algorithm can achieve an error $\varepsilon = 1/n^2$ with failure probability $\delta = 1/n^2$ in $\mathcal{O}(\mathrm{poly}(n))$ time, while, for the concept class $\mathcal{C}_n$ we consider, we can show that $\varepsilon = 1/2 - 1/\mathrm{poly}(n)$ and $\delta = 1/3$ would already break the DCR assumption.

3. Classical hardness

To show that classical learners cannot achieve significantly better than random guesses on the class C_n, we make use of a result from Alexi *et al.*[13]. This result makes use of the notion of a $\varepsilon(n)$-oracle:

Definition 3.4 ($\varepsilon(n)$-oracle)**.** *Let $O_{N,s}$ be a probabilistic oracle, such that $O_{N,s}(x)$ computes $g_{N,s}(x)$ correctly with probability $1/2 + \varepsilon(n)$ over the random choice of x and the internal randomness of the oracle. We say that $O_{N,s}$ is an $\varepsilon(n)$-oracle.*

Alexi *et al.* show that a $1/\mathrm{poly}(n)$-oracle is sufficient to break the DCR assumption.

Lemma 3.5 (Corollary (a) to Theorem 1 in [13])**.** *For any $g_{N,s} \in \mathcal{C}_n$, given a $1/poly(n)$-oracle $O_{N,s}$ to $g_{N,s}$, there exists a $\mathcal{O}(poly(n))$-time algorithm that uses $O_{N,s}$ to compute $g_N(x)$, $\forall x \in \mathbb{Z}_N$ (with success probability, e.g., $9/10$).*

We make use of this result to show the following lemma:

Lemma 3.6. *Under the discrete cube root assumption, no $\mathcal{O}(poly(n))$-time classical learning algorithm can achieve an expected error*

$$\Pr_{x\sim\mathcal{U}(\mathbb{Z}_N)}[h(x) \neq g_{N,s}(x)] \leq 1/2 - 1/poly(n) \tag{34}$$

with probability $2/3$ over the random generation of its training data and its internal randomness, and this for every concept $g_{N,s} \in \mathcal{C}_n$. $\mathcal{U}(\mathbb{Z}_N)$ is the uniform distribution over $\mathbb{Z}_N$.

Proof. Suppose by contradiction that such a learning algorithm would exist for a certain concept $g_{N,s} \in \mathcal{C}_n$. Then, given N, one can use this learning algorithm to generate with probability 2/3 a $1/\mathrm{poly}(n)$-oracle $O_{N,s} = h$. This is possible since the generation of training data for the concept $g_{N,s}$ is classically efficient given N. Now, by applying Lemma 3.5, one obtains a $\mathcal{O}(\mathrm{poly}(n))$-time algorithm that uses this $O_{N,s}$ to compute $g_N(x)$, $\forall x \in \mathbb{Z}_N$, with success probability 9/10. The overall success probability of this procedure, taken over the random choice of x, N and the learning algorithm is 0.6, which contradicts the DCR assumption. □

B. A simple shadow model

In this section we show how to construct a simple shadow model which can solve the same learning task for which we just showed classical hardness. This shadow model is obtained from the following flipped model:

$$f_{\boldsymbol{\theta}}(\boldsymbol{x}) = \mathrm{Tr}[\rho(\boldsymbol{\theta})O(\boldsymbol{x})]$$
$$\rho(\boldsymbol{\theta}) = |d', s'\rangle\langle d', s'| \ \& \ O(\boldsymbol{x}) = \sum_{d',s'} g_{N,s'}(\boldsymbol{x}) \, |d', s'\rangle\langle d', s'| \, . \tag{35}$$

That is, $\rho(\boldsymbol{\theta})$ consists of an n-qubit register which contains the candidate key $d' \in \mathbb{Z}_N$ and a second n-qubit register containing the candidate separating $s' \in \mathbb{Z}_N$ that is used to label whether $g_N(\boldsymbol{x}) = \boldsymbol{x}^d \bmod N \in [s', s' + \frac{N-1}{2}]$.

Lemma 3.7. *The concept class $\mathcal{C}_n$ is efficiently PAC learnable under the uniform distribution $\mathcal{U}(\mathbb{Z}_N)$ using a shadow model.*

Proof. We first describe how $\rho(\boldsymbol{\theta})$ can be computed efficiently quantumly. Using the specification of N provided by the training data[4], one can use Shor's algorithm to compute the factors p, q of N with arbitrarily high probability of success. This in turn allows to compute $\phi(N) = (p-1)(q-1)$ and the key $d' = d$ that satisfies $3d = 1 \bmod \phi(N)$ using Euclid's algorithm. As for the candidate separating s', it can be encoded using Pauli-X gates. The observable $O(\boldsymbol{x})$ can also be evaluated efficiently from computational basis measurements. For an outcome (d', s'), one simply computes $g_N(\boldsymbol{x}) = \boldsymbol{x}^d \bmod N$ via modular exponentiation and checks whether the output is in $[s', s' + \frac{N-1}{2}]$.
This model is naturally specified as a shadow model. One preparation of $\rho(\boldsymbol{\theta})$ followed by a computational basis measurement results in the classical advice $\omega(\boldsymbol{\theta}) = (d', s')$. The classical evaluation $\mathcal{A}(\boldsymbol{x}, \omega(\boldsymbol{\theta}))$ of a new data point $\boldsymbol{x}$ is done as explained in the last paragraph.
The only remaining learning aspect is to identify an s' close to s from a training set $\{(x, y_i = g_{N,s}(x))\}_{x\sim\mathcal{U}(\mathbb{Z}_N)}$. We show that a training set X of size $|X| \geq \frac{\log(\delta)}{\log(1-2\varepsilon)}$ is guaranteed to contain an x^* such that, for $s' = g_N(x^*)$, $|s - s'| \leq \varepsilon N$ with probability $1-\delta$. We take this x^* to be $x^* = \mathrm{argmin}_{x\in X}\mathcal{L}(x^d \bmod N)$, for $\mathcal{L}(y) = \sum_{x\in X} |g_{N,y}(x) - g_{N,s}(x)|$ the training loss on the training set X. We show this by proving:

$$\Pr\left(|s' - s| \geq \varepsilon N\right) \leq \delta. \tag{36}$$

This probability is precisely the probability that no $g_N(x) \in \{g_N(x)\}_{x\in X}$ is within ε distance of s, i.e.,

$$\Pr\left(\bigcap_{x\in X} g_N(x) \notin [s - \varepsilon N, s + \varepsilon N]\right). \tag{37}$$

As the elements of the training set are all identically distributed, we have that this probability is equal to

$$\Pr\left(g_N(x) \notin [s - \varepsilon N, s + \varepsilon N]\right)^{|X|}. \tag{38}$$

Since all the datapoints are uniformly sampled from $\mathbb{Z}_N$, the probability that a datapoint is in any region of size $2\varepsilon N$ is just 2ε. With the assumption that $|X| \geq \log_{1-2\varepsilon}(\delta)$ (and assuming $\varepsilon < 1/2$), we get:

$$\Pr\left(|s' - s| \geq \varepsilon N\right) \leq (1 - 2\varepsilon)^{\log_{1-2\varepsilon}(\delta/2)} = \delta. \tag{39}$$

From here, we simply notice that $|s' - s| \leq \varepsilon N$ guarantees an expected error

$$\Pr_{x\sim\mathcal{U}(\mathbb{Z}_N)}[g_{N,s'}(x) \neq g_{N,s}(x)] \leq 2\varepsilon. \tag{40}$$

□

[4] Strictly speaking, the PAC framework does not allow one to provide N explicitly in the training data. One way around this issue is to redefine the concepts to be of the form $\tilde{g}_{N,s} : \{0,1\} \times \mathbb{Z}_N \to \{0,1\} \mid \tilde{g}_{N,s}(0, x) = g_{N,s}(x)$ and $\tilde{g}_{N,s}(1, x) =$ "j-th bit of N, for j encoded in the first $\log(N)$ bits of x". This way, we can efficiently recover N from the uniform distribution $\mathcal{U}(\{0,1\} \times \mathbb{Z}_N)$, while only marginally impacting the training data.

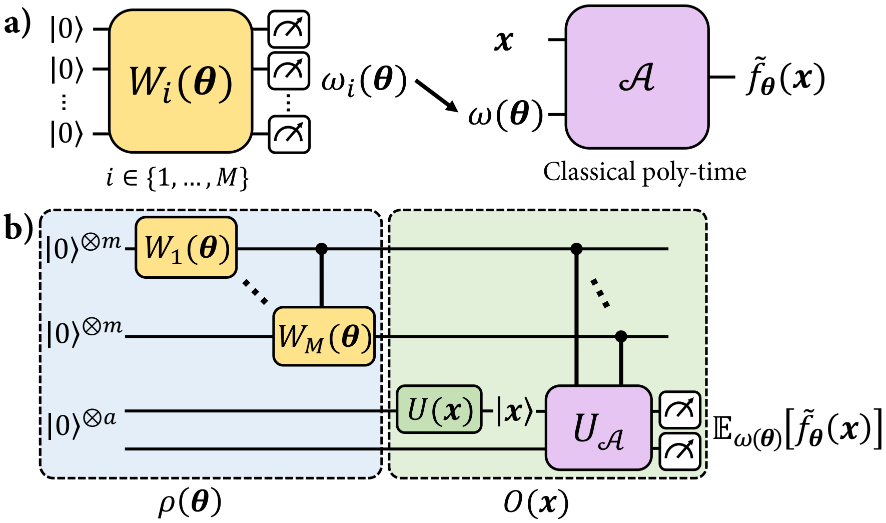

Supplementary Figure 2. **All shadow models can be expressed as shadowfiable flipped models**. a) A shadow model consists of M unitary circuits $W_i(\boldsymbol{\theta})$ that can be chosen adaptively, and that generate advice $\omega_i(\theta)$ from computational basis measurements of the states $W_i(\boldsymbol{\theta})\,|0\rangle^m$. This advice, along with a (binary description of) an input $\boldsymbol{x} \in \mathbb{R}^d$ are processed by a classical algorithm $\mathcal{A}$ to compute an approximation $\widetilde{f}_{\boldsymbol{\theta}}$ of the shadowfiable model $f_{\boldsymbol{\theta}}$. b) A coherent implementation of this shadow model, where the unitaries $W_i(\boldsymbol{\theta})$ are applied on different m-qubit registers, and coherently controlled by previous registers (for adaptivity). These M registers constitute the coherent encoding of the advice $|\omega(\boldsymbol{\theta})\rangle$. The algorithm $\mathcal{A}$ can then be simulated by a reversible quantum computation $U_{\mathcal{A}}$ (see Sec. 3.2.5. in [14]) that processes a binary encoding $|\boldsymbol{x}\rangle$ of $\boldsymbol{x}$ and the coherent advice $|\omega(\boldsymbol{\theta})\rangle$ (either directly or indirectly via controlled operations that imprint $|\omega(\boldsymbol{\theta})\rangle$ on an auxiliary register). This coherent implementation of the shadow model can be viewed as a shadowfiable flipped model $g_{\boldsymbol{\theta}}(\boldsymbol{x}) = \mathrm{Tr}[\rho(\boldsymbol{\theta})O(\boldsymbol{x})]$, such that one evaluation of this model samples an advice $\omega(\boldsymbol{\theta})$ and evaluates $\mathcal{A}(\boldsymbol{x}, \omega(\boldsymbol{\theta}))$ for that advice and a given $\boldsymbol{x}$.

4. Relations between shadow models

A. Flipped models are universal

In this section we show that any shadowfiable model as defined in Defs. 2 and 3 in the main text can be approximated by an efficiently shadowfiable flipped model. This result corresponds to Lemma 4 in the main text, and more formally in the following lemma.

Lemma 4.1. *Let $f_{\boldsymbol{\theta}}$ be a shadowfiable model acting on n qubits as defined in Def. 3 in the main text and let $\varepsilon, \delta > 0$. There exists a flipped model $g_{\boldsymbol{\theta}}(\boldsymbol{x}) = \mathrm{Tr}[\rho(\boldsymbol{\theta})O(\boldsymbol{x})]$ acting on $\mathcal{O}(poly(n))$ qubits and evaluatable in $\mathcal{O}(poly(n, 1/\varepsilon))$ time such that*

$$\max_{\boldsymbol{x}\in\mathcal{X}} |f_{\boldsymbol{\theta}}(\boldsymbol{x}) - g_{\boldsymbol{\theta}}(\boldsymbol{x})| \leq \varepsilon. \tag{41}$$

Moreover, this flipped model is also shadowfiable for the error parameter ε and the success probability $1-\delta$. More precisely, a computational basis measurement of $\rho(\boldsymbol{\theta})$ yields an advice $\omega(\boldsymbol{\theta})$ such that with probability $1-\delta$ over the randomness of this measurement, we have

$$\max_{\boldsymbol{x}\in\mathcal{X}} |f_{\boldsymbol{\theta}}(\boldsymbol{x}) - \widetilde{g}_{\boldsymbol{\theta}}(\boldsymbol{x})| \leq \varepsilon, \tag{42}$$

where $\widetilde{g}_{\boldsymbol{\theta}}(\boldsymbol{x}) = \mathcal{A}(\boldsymbol{x}, \omega(\boldsymbol{\theta}))$ is a classical $\mathcal{O}(poly(n, 1/\varepsilon, 1/\delta, d))$-time algorithm that processes the advice $\omega(\boldsymbol{\theta})$ along with an input $\boldsymbol{x} \in \mathbb{R}^d$.

Proof. By Def. 3 in the main text, the shadowfiable model $f_{\boldsymbol{\theta}}$ admits for every error of approximation $\varepsilon' > 0$ and probability of failure $\delta' > 0$ a shadow model $\widetilde{f}_{\boldsymbol{\theta}}$ that uses $m \cdot M \in \mathcal{O}(\mathrm{poly}(n, 1/\varepsilon', 1/\delta'))$ qubits and guarantees

$$\max_{\boldsymbol{x}\in\mathcal{X}} \left| f_{\boldsymbol{\theta}}(\boldsymbol{x}) - \widetilde{f}_{\boldsymbol{\theta}}(\boldsymbol{x}) \right| \leq \varepsilon' \tag{43}$$

with probability $1-\delta'$ over the generation of its advice. Out of this shadow model, we use the construction described in Supplementary Figure 2.b) to define the flipped model $g_{\boldsymbol{\theta}}(\boldsymbol{x})$. Since this flipped model corresponds to the evaluation of the shadow model $\widetilde{f}_{\boldsymbol{\theta}}(\boldsymbol{x})$ averaged over the randomness of $\omega(\boldsymbol{\theta})$, we have, for all $\boldsymbol{x} \in \mathbb{R}^d$:

$$|f_{\boldsymbol{\theta}}(\boldsymbol{x}) - g_{\boldsymbol{\theta}}(\boldsymbol{x})| \leq \left|(1-\delta')\varepsilon' + \delta'\left\|\widetilde{f}_{\boldsymbol{\theta}}\right\|\right| \tag{44}$$

$$\leq \varepsilon' + \delta'\left(\left\|\widetilde{f}_{\boldsymbol{\theta}}\right\| + \varepsilon'\right) \tag{45}$$

where we use that with probability $1-\delta'$ we have $\left|f_{\boldsymbol{\theta}}(\boldsymbol{x}) - \widetilde{f}_{\boldsymbol{\theta}}(\boldsymbol{x})\right| \leq \varepsilon'$ and otherwise assume the worse case error $\left|f_{\boldsymbol{\theta}}(\boldsymbol{x}) - \widetilde{f}_{\boldsymbol{\theta}}(\boldsymbol{x})\right| \leq 2\left\|\widetilde{f}_{\boldsymbol{\theta}}\right\|$, for $\left\|\widetilde{f}_{\boldsymbol{\theta}}\right\| = \max_{\boldsymbol{x}\in\mathcal{X}} \left|\widetilde{f}_{\boldsymbol{\theta}}(\boldsymbol{x})\right|$ (which can also be capped to $\max_{\boldsymbol{x}\in\mathcal{X}} |f_{\boldsymbol{\theta}}(\boldsymbol{x})|$ without loss of generality). Therefore, by setting $\varepsilon' = \frac{\varepsilon}{2}$ and $\delta' = \min\left\{\frac{\varepsilon}{2\left(\left\|\widetilde{f}_{\boldsymbol{\theta}}\right\| + \varepsilon'\right)}, \delta\right\}$ (important for the second part of the proof), we get

$$\max_{\boldsymbol{x}\in\mathcal{X}} |f_{\boldsymbol{\theta}}(\boldsymbol{x}) - g_{\boldsymbol{\theta}}(\boldsymbol{x})| \leq \varepsilon. \tag{46}$$

This proves the first part of the lemma. From here, it is straightforward to notice that a measurement of $\rho(\boldsymbol{\theta}) = |\omega(\boldsymbol{\theta})\rangle\langle\omega(\boldsymbol{\theta})| \otimes |0\rangle\langle 0|^{\otimes a}$ in the computational basis yields an advice $\omega(\boldsymbol{\theta})$ such that the algorithm $\mathcal{A}$ associated to the shadow model $\widetilde{f}_{\boldsymbol{\theta}}$ satisfies

$$\max_{\boldsymbol{x}\in\mathcal{X}} |f_{\boldsymbol{\theta}}(\boldsymbol{x}) - \mathcal{A}(\boldsymbol{x}, \omega(\boldsymbol{\theta}))| \leq \varepsilon' < \varepsilon \tag{47}$$

with probability at least $1-\delta' \geq 1-\delta$ over the randomness of measuring the advice. □

B. BQP and P/poly

In this section we give a rigorous proof that there exist quantum models that are not shadowfiable, under the assumption that $\mathsf{BQP} \not\subset \mathsf{P/poly}$. The approach we take is similar to that of Huang *et al.* [1] (Appendix A), although we consider different complexity classes and therefore show different results. Let us start by noting that the shadow models defined in Def. 2 compute functions in a subclass of $\mathsf{P/poly}$, namely the subclass in which the advice $\omega(\boldsymbol{\theta})$ is efficiently generated from the measurements of a polynomial number of quantum circuits. We call this complexity class $\mathsf{BPP/qgenpoly}$ and it is obvious that $\mathsf{BPP/qgenpoly} \subseteq \mathsf{P/poly}$ as the latter is equal to $\mathsf{BPP/poly}$ [4] and contains all classically efficiently computable functions with advice of polynomial length, without any constraints on how the advice is generated.

On the other hand, we know that quantum models can compute all functions in BQP. To see this, we first refer the reader to the definition of BQP in Def. 1.4. It is easy to show that for any language L in BQP, there exists a quantum model which can decide this language. Consider the following quantum model $f_n = \mathrm{Tr}[\rho(x)O_n]$, depicted in Supplementary Figure 3:

$$\rho(x) = \bigotimes_{i=1}^{n} X_i^{x_i} |0\rangle\langle 0| X_i^{x_i} \quad \& \quad O_n = U_n^\dagger Z_1 U_n \tag{48}$$

Where X_i is the Pauli-X gate acting on the i-th qubit, here parametrized by $x_i \in \{0,1\}$, the i-th bit of x, Z_1 is the Pauli observable on the first qubit, and U_n is the quantum circuit used to decide the language L in Def. 1.4. Then:

1. For all $x \in L$, $f_n(x) = \Pr$[the output of $U_{|x|}$ applied on the input x is 1] - $\Pr$[the output of $U_{|x|}$ applied to the input x is 0] $\geq 2/3 - 1/3 = 1/3$.
2. For all $x \notin L$, $f_n(x) = \Pr$[the output of $U_{|x|}$ applied on the input x is 1] - $\Pr$[the output of $U_{|x|}$ applied to the input x is 0] $\leq 1/3 - 2/3 = -1/3$.

Therefore, as $f_n(x) > 0$ if $x \in L$ and $f_n(x) < 0$ if $x \notin L$ such quantum model could efficiently decide the language. We show that if all such quantum models would be shadowfiable then $\mathsf{BQP} \subseteq \mathsf{P/poly}$.

Lemma 4.2. *If all quantum models $f_{\boldsymbol{\theta}}$ are shadowfiable with the guarantee that, $\forall x \in \mathcal{X}$,*

$$\left|f_{\boldsymbol{\theta}}(x) - \widetilde{f}_{\boldsymbol{\theta}}(x)\right| < 0.15, \tag{49}$$

with probability at least $2/3$ over the shadowing phase and the randomness of evaluating the shadow model $\widetilde{f}_{\boldsymbol{\theta}}$, then $\mathsf{BQP} \subseteq \mathsf{P/poly}$.

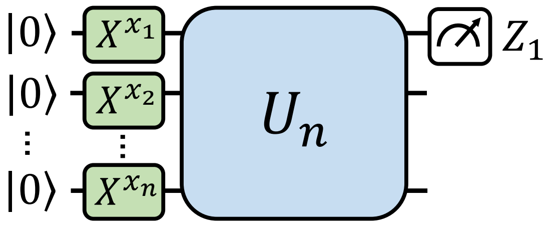

Supplementary Figure 3. **A universal quantum model for BQP**. For an n-dimensional input $\boldsymbol{x} \in \{0,1\}^n$, this model acts on n qubits, encodes $\boldsymbol{x}$ in its binary form $|\boldsymbol{x}\rangle$ and applies a poly(n)-time unitary U_n before a Pauli-Z measurement of the first qubit. For appropriately chosen unitaries $\{U_n : n \in \mathbb{N}\}$, this model can decide any language in BQP. For more general computational basis measurements, the resulting model can represent arbitrary functions in FBQP, the functional version of BQP.

Proof. Consider a language L in BQP. We showed that there exists a quantum model $f_{\boldsymbol{\theta}} = f_n$ which can determine, for every $x \in \{0,1\}^n$, whether x is in L. Now, by our assumption, there exists a shadow model $\widetilde{f}_n$ such that, for every $x \in \{0,1\}^n$, $|f_n(x) - \widetilde{f}_n(x)| < 0.15$ with probability greater than 2/3 over the randomness of sampling the advice $\omega(\boldsymbol{\theta})$ and the (potential) internal randomness of its classical algorithm $\mathcal{A}$. To get rid of these two sources of randomness, we make use of the same proof strategy as for Adleman's theorem [4]: Consider now a new algorithm $\mathcal{A}'$ which runs $\mathcal{A}$ $18n$ times, each time using a new sampled advice string $\omega(\boldsymbol{\theta})$ and a new random bit-string for its internal randomness. Then we take a majority vote from the $18n$ runs. By Chernoff bound, the probability that for any $x \in \{0,1\}^n$ the algorithm $\mathcal{A}'$ fails to determine if x belongs to L is at most $1/e^n$. Then, by union bound, the probability that $\mathcal{A}'$ decides all the $x \in \{0,1\}^n$ correctly is at least $1 - 2^n/e^n > 0$. This implies that there exists a particular choice of the $18n$ strings $\omega(\boldsymbol{\theta})$ and $18n$ random bit-strings used in each run of the algorithm $\mathcal{A}$ such that $\mathcal{A}'$ is correct for all x. The algorithm $\mathcal{A}'$, along with this particular choice of $18n$ strings as advice (which is of size polynomial in n) is our P/poly algorithm. Note that it guarantees $\forall \boldsymbol{x} \in \{0,1\}^n$, $\widetilde{f}_n(x) > 0$ if $x \in L$ and $\widetilde{f}_n(x) < 0$ if $x \notin L$. Therefore we can use the sign of $\widetilde{f}_n(x)$ to determine whether $x \in L$. This implies BQP $\subseteq$ P/poly. □

C. Shadow models beyond Fourier

1. Generalization to the full domain $\boldsymbol{x} \in \mathbb{R}^n$

In the main text, we showed how the model:

$$
\begin{aligned}
f_{\boldsymbol{y}}(\boldsymbol{x}) &= \mathrm{Tr}[\rho(\boldsymbol{x})O(\boldsymbol{y})] \\
\rho(\boldsymbol{x}) &= \bigotimes_{i=1}^{n} R_Y(x_i)\,|0\rangle\langle 0|\,R_Y^\dagger(x_i) \ \&\ O(\boldsymbol{y}) = |\boldsymbol{y}\rangle\langle \boldsymbol{y}|\,.
\end{aligned} \tag{50}
$$

for $\boldsymbol{y} \in \{0,1\}^{\otimes n}$ is not efficiently shadowfiable when we restrict the domain of $\boldsymbol{x}$ to be $\{0,\pi\}^n$ as, on this domain, $f_{\boldsymbol{y}}(\boldsymbol{x}) = \delta_{\boldsymbol{x}/\pi,\boldsymbol{y}}$ plays the role of a database search oracle. We can extend this intractability result to the full domain $\boldsymbol{x} \in \mathbb{R}^n$ by that noting that more general encoding states (even more general than those in Eq. (50)) take the form

$$
\rho(\boldsymbol{x}) = |\psi(\boldsymbol{x})\rangle\langle\psi(\boldsymbol{x})|\,, \quad \text{for} \quad |\psi(\boldsymbol{x})\rangle = \sum_{\boldsymbol{j}=1}^{2^n} \alpha_{\boldsymbol{j}}(\boldsymbol{x})\,|\boldsymbol{j}\rangle \tag{51}
$$

where $\alpha_{\boldsymbol{j}}(\boldsymbol{x}) \in \mathbb{C}$ is an arbitrary amplitude associated to a computational basis state $|\boldsymbol{j}\rangle$.

Now note that when we evaluate the quantum model $f_{\boldsymbol{y}}(\boldsymbol{x})$ on a quantum computer, we do not have direct access to the expectation values $\mathrm{Tr}[\rho(\boldsymbol{x})O(\boldsymbol{y})]$ it corresponds to. We rather sample an eigenvalue of $O(\boldsymbol{y})$ according to the Born rule applied to $\rho(\boldsymbol{x})$. Therefore, a single evaluation of $f_{\boldsymbol{y}}(\boldsymbol{x})$ for the encoding $\rho(\boldsymbol{x})$ defined in Eq. (51) returns 1 with probability $|\alpha_{\boldsymbol{y}}(\boldsymbol{x})|^2$ and 0 otherwise.

Let us call $U_{\boldsymbol{y}}$ the Grover operator associated to the database search oracle that marks $\boldsymbol{y}$, i.e.,

$$
U_{\boldsymbol{y}} : |\boldsymbol{j}\rangle\,|0\rangle \mapsto |\boldsymbol{j}\rangle\,|\delta_{\boldsymbol{j},\boldsymbol{y}}\rangle\,. \tag{52}
$$

and apply it on the state $|\psi(\boldsymbol{x})\rangle|0\rangle$:

$$U_{\boldsymbol{y}}|\psi(\boldsymbol{x})\rangle|0\rangle = \sum_{\boldsymbol{j}=1}^{2^n} \alpha_{\boldsymbol{j}}(\boldsymbol{x})|\boldsymbol{j}\rangle|\delta_{\boldsymbol{j},\boldsymbol{y}}\rangle. \tag{53}$$

One can then notice that measuring the second register also yields 1 with probability $|\alpha_{\boldsymbol{j}}(\boldsymbol{x})|^2$ and 0 otherwise. Therefore, a single evaluation of $f_{\boldsymbol{y}}(\boldsymbol{x})$ is as powerful as querying the Grover operator oracle in superposition, which still suffers from the same query complexity lower bound as querying it classically.

2. Fourier decomposition

In this subsection, we derive the Fourier-series decomposition of the model in Eq. (50), i.e., the frequency spectrum Ω and the Fourier coefficients $c_{\boldsymbol{\omega}}$ in the expression:

$$f_{\boldsymbol{y}}(\boldsymbol{x}) = \sum_{\boldsymbol{\omega}\in\Omega} c_{\boldsymbol{\omega}}(\boldsymbol{y})e^{-i\boldsymbol{\omega}\cdot\boldsymbol{x}}. \tag{54}$$

We start by noting that the model has a product structure, in which each (x_i, y_i) pair contributes similarly. Notably, the overlap between the i-th qubit in the state $R_Y(x_i)|0\rangle = \cos\left(\frac{x_i}{2}\right)|0\rangle - \sin\left(\frac{x_i}{2}\right)|1\rangle$ and the state $|y_i\rangle$ is:

$$\left|\left(\cos\left(\frac{x_i}{2}\right)\langle 0| - \sin\left(\frac{x_i}{2}\right)\langle 1|\right)|y_i\rangle\right|^2 = \cos^2\left(\frac{x_i + \pi y_i}{2}\right). \tag{55}$$

From the Euler decomposition of $\cos(x) = \frac{e^{ix}+e^{-ix}}{2}$, we get:

$$\cos^2\left(\frac{x_i + \pi y_i}{2}\right) = \frac{2 + e^{i(x_i+\pi y_i)} + e^{-i(x_i+\pi y_i)}}{4}, \tag{56}$$

such that

$$f_{\boldsymbol{y}}(\boldsymbol{x}) = \prod_{i=1}^{n} \frac{2 + e^{i(x_i+\pi y_i)} + e^{-i(x_i+\pi y_i)}}{4} \tag{57}$$

$$= \sum_{\boldsymbol{\omega}\in\{-1,0,1\}^n} \frac{1}{2^{n+|\boldsymbol{\omega}|}} e^{i(\boldsymbol{x}+\pi\boldsymbol{y})\cdot\boldsymbol{\omega}} \tag{58}$$

$$= \sum_{\boldsymbol{\omega}\in\{-1,0,1\}^n} \frac{e^{i\pi\boldsymbol{y}\cdot\boldsymbol{\omega}}}{2^{n+|\boldsymbol{\omega}|}} e^{i\boldsymbol{x}\cdot\boldsymbol{\omega}} \tag{59}$$

where $|\boldsymbol{\omega}| = \sum_{i=1}^{n}|\omega_i|$. We can therefore identify $\Omega = \{-1,0,1\}^n$ and $c_{\boldsymbol{\omega}}(\boldsymbol{y}) = \frac{e^{i\pi\boldsymbol{y}\cdot\boldsymbol{\omega}}}{2^{n+|\boldsymbol{\omega}|}}$ and note that the frequency spectrum of this model is exponentially large in n, with all its coefficients being non-zero, exponentially small in n, and differing only by a phase depending on $\boldsymbol{y}$. This justifies the exponential sample complexity needed to identify $\boldsymbol{y}$.

3. Obfuscation of $\boldsymbol{y}$

At this point, the interested reader might also point out that the obfuscation of the marked basis state $|\boldsymbol{y}\rangle\langle\boldsymbol{y}|$ only occurs somehow artificially by considering the observable $O(\boldsymbol{y})$ as a black-box. That is, we consider as a black-box not only the input-independent gates in the circuit but also the mapping from computational basis state to real values when measuring the output state of the circuit. More naturally, when measuring a basis state $|\boldsymbol{j}\rangle$, one would have a computable function that returns its corresponding eigenvalue, which in this case could reveal $\boldsymbol{y}$. An easy fix for this is to include the encoding of $\boldsymbol{y}$ in the circuit, by redefining $O(\boldsymbol{y})$ as

$$O(\boldsymbol{y}) = \bigotimes_{i=1}^{n} X^{y_i}|0\rangle\langle 0|X^{y_i}, \tag{60}$$

which delegates the obfuscation of $\boldsymbol{y}$ to gates in the circuit.

One can also go a step further in this obfuscation by considering instead the following observables

$$O(\boldsymbol{y}) = V_{\mathrm{DLP}}\,|\boldsymbol{y}\rangle\langle\boldsymbol{y}|\,V_{\mathrm{DLP}}^{\dagger} \tag{61}$$

where V_{DLP} is the unitary[5] that maps a basis state to its discrete logarithm:

$$V_{\mathrm{DLP}} : |\boldsymbol{y}\rangle \mapsto \left|(\log_g(\boldsymbol{y}) \text{ mop } p) + 1\right\rangle = |\boldsymbol{y}'\rangle\,. \tag{62}$$

Now, even the knowledge of $\boldsymbol{y}$ and a description of the quantum circuit do not help identify $\boldsymbol{y}'$ classically, under the classical hardness assumption of DLP. Moreover, we still retain the hardness of Fourier-shadowing the resulting model $f_{\boldsymbol{y}}(\boldsymbol{x}) = \mathrm{Tr}[\rho(\boldsymbol{x})O(\boldsymbol{y})]$ from the same database-search arguments. And finally, the flipped model associated to $f_{\boldsymbol{y}}$ still benefits from the same efficient shadowing procedure, as $O(\boldsymbol{y})$ can be prepared on a quantum computer and measured in the computational basis to reveal $\boldsymbol{y}'$.

D. Shadowfiablility

In our definition of shadowfiable models (see Def. 3 in the main text), we take the convention that the shadow model should agree with the original quantum model for all possible inputs $\boldsymbol{x} \in \mathcal{X}$. This choice makes sense for two reasons:

1. We would like the shadowing procedure to work on all potential data distributions, as to be applicable in all learning tasks a given quantum model could be used in.

2. In the context of machine learning, one typically considers PAC conditions, meaning that the final model should achieve a small error $\mathbb{E}_{\boldsymbol{x}\sim\mathcal{D}}|h(\boldsymbol{x}) - g(\boldsymbol{x})|$ only with respect to some data distribution $\mathcal{D}$. Note that if the quantum model to be shadowfied achieves these PAC conditions, our demands on worst-case approximation will guarantee that the shadow model achieves them as well.

Nonetheless, one may still be interested in a notion of shadowfiability that considers an average-case error

$$\mathbb{E}_{\boldsymbol{x}\sim\mathcal{D}}\left|\widetilde{f}_{\boldsymbol{\theta}}(\boldsymbol{x}) - f_{\boldsymbol{\theta}}(x)\right| \tag{63}$$

with respect to a specified data distribution $\mathcal{D}$. It is not entirely clear which models can still be shadowfied in this way. But our results on the universality of flipped models (Lemma 4 in the main text and Lemma 4.1 in the Appendix), as well as on the existence of quantum models that are not shadowfiable (Theorem 5 in the main text and Lemma 4.2) would also hold. More precisely, for each of these results, respectively:

1. The same proof structure of Lemma 4.1 can be used, as the constructed flipped model only adds a small controllable error to each $\boldsymbol{x} \in \mathcal{X}$.

2. One can consider here instead of quantum models that compute arbitrary functions in BQP, a restricted model that computes (single bits of) the discrete logarithm $\log_g(\boldsymbol{x})$ mod p (analogous to our DCR concept class defined in Eq. (32)). The result of Liu *et al.* [15] (Theorem 6 in the Supplementary Information) shows the classical hardness of achieving an expected error $\mathbb{E}_{\boldsymbol{x}\sim\mathcal{D}}|h(\boldsymbol{x}) - g(\boldsymbol{x})| \leq 1/2 - 1/\mathrm{poly}(n)$ for such target functions (and a hypothesis h producing labels $h(\boldsymbol{x}) \in \{0, 1\}$), under the assumption that $\mathsf{DLP} \notin \mathsf{BPP}$. One can then use this result to show that there exist quantum models that are not average-case shadowfiable under the assumption that $\mathsf{DLP} \notin \mathsf{P/poly}$.

---

[5] Note that this is indeed a unitary transformation, but that potentially needs auxiliary qubits to be implemented unitarily on a quantum computer.

[1] H.-Y. Huang, M. Broughton, M. Mohseni, R. Babbush, S. Boixo, H. Neven, and J. R. McClean, Power of data in quantum machine learning, Nature Communications **12**, 1 (2021).

[2] L. Leone, S. F. Oliviero, and A. Hamma, Nonstabilizerness determining the hardness of direct fidelity estimation, Physical Review A **107**, 022429 (2023).

[3] H.-Y. Huang, M. Broughton, J. Cotler, S. Chen, J. Li, M. Mohseni, H. Neven, R. Babbush, R. Kueng, J. Preskill, *et al.*, Quantum advantage in learning from experiments, Science **376**, 1182 (2022).

[4] L. Adleman, Two theorems on random polynomial time, in *19th Annual Symposium on Foundations of Computer Science (sfcs 1978)* (IEEE Computer Society, 1978) pp. 75–83.

[5] P. Dagum, R. Karp, M. Luby, and S. Ross, An optimal algorithm for monte carlo estimation, SIAM Journal on computing **29**, 1484 (2000).

[6] R. Canetti, G. Even, and O. Goldreich, Lower bounds for sampling algorithms for estimating the average, Information Processing Letters **53**, 17 (1995).

[7] S. Aaronson, The learnability of quantum states, Proceedings of the Royal Society A: Mathematical, Physical and Engineering Sciences **463**, 3089 (2007).

[8] M. Anthony and P. L. Bartlett, Function learning from interpolation, Combinatorics, Probability and Computing **9**, 213 (2000).

[9] A. Ambainis, A. Nayak, A. Ta-Shma, and U. Vazirani, Dense quantum coding and quantum finite automata, Journal of the ACM (JACM) **49**, 496 (2002).

[10] L. Wang and X. Ma, Bounds of graph energy in terms of vertex cover number, Linear Algebra and its Applications **517**, 207 (2017).

[11] S. Jerbi, L. J. Fiderer, H. Poulsen Nautrup, J. M. Kübler, H. J. Briegel, and V. Dunjko, Quantum machine learning beyond kernel methods, Nature Communications **14**, 517 (2023).

[12] M. J. Kearns and U. Vazirani, *An introduction to computational learning theory* (MIT press, 1994).

[13] W. Alexi, B. Chor, O. Goldreich, and C. P. Schnorr, Rsa and rabin functions: Certain parts are as hard as the whole, SIAM Journal on Computing **17**, 194 (1988).

[14] M. A. Nielsen and I. L. Chuang, *Quantum Computation and Quantum Information* (Cambridge University Press, 2000).

[15] Y. Liu, S. Arunachalam, and K. Temme, A rigorous and robust quantum speed-up in supervised machine learning, Nature Physics , 1 (2021).
